# Single-cell characterization of malignant phenotypes and microenvironment alteration in retinoblastoma

**DOI:** 10.1038/s41419-022-04904-8

**Published:** 2022-05-06

**Authors:** Cheng Wu, Jiaqi Yang, Wei Xiao, Zehang Jiang, Shuxia Chen, Dianlei Guo, Ping Zhang, Chunqiao Liu, Huasheng Yang, Zhi Xie

**Affiliations:** State Key Laboratory of Ophthalmology, Zhongshan Ophthalmic Center, Sun Yat-sen University, Guangzhou, China

**Keywords:** Eye cancer, Transcriptomics, Cancer microenvironment, Tumour heterogeneity

## Abstract

Retinoblastoma (RB) is the most common primary intraocular malignancy of childhood. It is known that the tumor microenvironment (TME) regulates tumorigenesis and metastasis. However, how the malignant progression in RB is determined by the heterogeneity of tumor cells and TME remains uncharacterized. Here, we conducted integrative single-cell transcriptome and whole-exome sequencing analysis of RB patients with detailed pathological and clinical measurements. By single-cell transcriptomic sequencing, we profiled around 70,000 cells from tumor samples of seven RB patients. We identified that the major cell types in RB were cone precursor-like (CP-like) and MKI67+ cone precursor (MKI67+ CP) cells. By integrating copy number variation (CNV) analysis, we found that RB samples had large clonal heterogeneity, where the malignant MKI67+ CP cells had significantly larger copy number changes. Enrichment analysis revealed that the conversion of CP-like to MKI67+ CP resulted in the loss of photoreceptor function and increased cell proliferation ability. The TME in RB was composed of tumor-associated macrophages (TAMs), astrocyte-like, and cancer-associated fibroblasts (CAFs). Particularly, during the invasion process, TAMs created an immunosuppressive environment, in which the proportion of TAMs decreased, M1-type macrophage was lost, and the TAMs-related immune functions were depressed. Finally, we identified that TAMs regulated tumor cells through GRN and MIF signaling pathways, while TAMs self-regulated through inhibition of CCL and GALECTIN signaling pathways during the invasion process. Altogether, our study creates a detailed transcriptomic map of RB with single-cell characterization of malignant phenotypes and provides novel molecular insights into the occurrence and progression of RB.

## Introduction

Retinoblastoma (RB) is the most common primary intraocular malignancy of childhood [1, 2], where eye containing RB tumor that display threatening clinical features may be surgically removed [3, 4]. RB is generally caused by mutations in the tumor suppressor gene (RB1) encoded protein, pRB [5]. Tumor cells in RB are highly heterogeneous and understanding intratumoral heterogeneity will facilitate a better understanding of therapy responses and treatment resistance in cancers [6, 7]. McEvoy et al. [8] reported that human and mouse RBs had molecular, cellular, and neurochemical features of multiple cell classes, including amacrine or horizontal interneurons, retinal progenitor cells (RPC) and photoreceptors. Moreover, recent studies suggested that the tumor microenvironment (TME) could regulate tumorigenesis and metastasis in a variety of tumors [9, 10]. Previous studies revealed the presence of stromal cell types in TME of RB using immunohistochemistry [11]. It also has been reported that retinal astrocytes enhanced the proliferation of cone-like RB cells by deploying IGFBP-5 [12], while macrophages facilitated tumor development by expression of growth factors [13]. However, how tumor and immune cells determine malignant progression in RB remains uncharacterized.

Recent advances in single-cell RNA sequencing (scRNA-seq) effectively have revealed intratumoral heterogeneity, rare subpopulation, and cell of origin in human cancers, including primary glioblastoma [14], head and neck cancer [15], renal tumors [16], breast cancer [17] and pancreatic ductal adenocarcinoma [18, 19]. Besides, scRNA-seq also has provided many critical insights into TME. For example, Zheng et al. [20] revealed landscape of infiltrating T cells in liver cancer and Azizi et al. [21] built an immune map of breast cancer, revealing continuous T cell activation and differentiation states. More recently, attempts also have been made to understand cellular heterogeneity and origins of RB using scRNA-seq. Collin et al. [22] revealed G2/M cone precursors as the cell of origin in RB. Yang et al. [23] revealed that the RB cells originated from the cell cycle-assoicated cone precursors. However, due to the limited number of cells (8086/14,739) and samples (two samples) in these two studies, how the malignant progression in RB is determined by the heterogeneity of tumor cells and TME remains uncharacterized.

Here, we applied scRNA-seq technology to seven RB primary tumors with pathological and clinical measurements. The transcriptomic profiles contained a total of 69,820 cells, mainly including cone precursor (CP)-like subtypes, MKI67+ CP subtypes, and glial cells (astrocyte-like and TAMs). Copy number variation (CNV) analysis revealed the malignant MKI67 + CP cells had significantly larger copy number changes. We further showed that the conversion of CP-like to MKI67+ CP caused the loss of photoreceptor function and increased cell proliferation ability in RB. Moreover, we found that during the invasion process, TAMs created an immunosuppressive environment, where the proportion of TAMs decreased, M1-type macrophage was lost. Finally, we demonstrated that TAMs directly regulated tumor cells through GRN and MIF signaling pathways, while regulated themselves through inhibition of CCL and GALECTIN signaling pathways during the invasion process. Our study presents a comprehensive and rich single-cell transcriptome landscape of human RB and provides molecular insights into RB progression.

## Results

### Single-cell atlas in RB

**Fig. 1:**
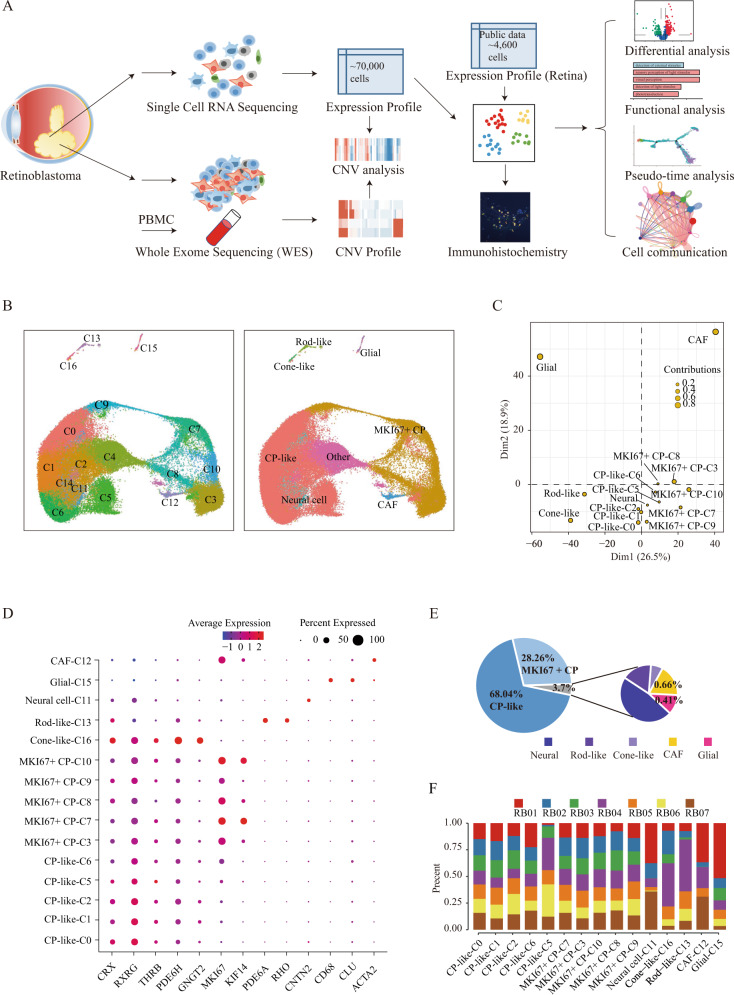
Single-cell expression map in RB. **A** Overview of workflow. **B** U-MAP visualization of cell clusters (left) and cell types (right) in RB. **C** PCA of mean gene expression of cell types in RB. **D** Expression heatmap of known marker genes in RB. **E** The proportion of cells of different cell types in RB; **F** representation of the seven donor RB samples in each cell type.

Using 10× Genomics, we isolated and sequenced RB cells from whole tumor suspensions of seven RB samples after eyeball removal surgery (Fig. 1A, Table S1), where 69,820 cells were taken forward for further analysis after preprocessing, quality control and batch effect removal (Table S2 and Methods). Correlation analysis showed high consistency among the mean gene expression profile of the technically repeated sequencing samples (*p* < 1e^−16^, *r* > 0.99, Pearson correlation) (Fig. S1A–C), and high correlation for the single-cell expression profile between different samples (*p* < 1e^−16^, average r of 0.96, Pearson correlation) (Fig. S1D). Additionally, we found that the expression levels of genes including many known RB-related oncogenes or suppressor genes, such as RB1, MDM4, MKI67 and TFF1, were consistent between our single-cell and bulk-seq expression profiles (GSE111168), with a high correlation (*p* < 1e^−16^, *r* = 0.72, Pearson correlation) (Fig. S1E), indicating that our single-cell datasets were highly reproduceable and reliable.

Graph-based clustering using the first 30 principal components showed that the cells were distributed into 17 distinct clusters (C0-C16) (Fig. S2A), ranging in size from 1,385 to 2,972 genes on average (Fig. S2B) and from 157 to 11,222 cells (Fig. S2C) for each cluster. Notably, each cluster was composed of cells from multiple samples (Fig. 1B, F), demonstrating a better data integration. To identify the cell types in RB, we manually curated a set of known retinal cell-type specific markers to characterize each cluster (Table S3). We used three sets of marker genes. (1) Marker genes of immature precursors including CRX, which is specific to cone, rod, and bipolar cells (BCs); RXRG, which is associated with cone and retinal ganglion cells (RGCs); THRB, which is specific to early cone photoreceptors. (2) Marker genes of mature photoreceptors including RHO and PDE6A, which are expressed by rods; ARR3 and GNGT2, which are specific to cones. (3) Marker genes of retinal neurons-related cells including POU4F2, NEFL, SNCG, ATOH7, EBF3, THY1 and NRN1, which are highly expressed in RGC; ONECUT1, ONECUT2, ONECUT3, LHX1 and TFAP2B, which are associated with horizontal cells (HC); VSX2, VSX1 and TRPM1, which are expressed by BC; GAD1, CALB1, NRXN2, TFAP2A and PROX, which are specific to amacrine cells (AC) [24–29].

We found that most of the clusters were characterized by high expression of the markers of CP (CRX, RXRG, THRB) (Figs. 1D, S2C). Notably, five clusters, including C3, C7, C8, C9, and C10, not only expressed the markers of cone precursors, but also highly expressed the proliferation-related genes (MKI67, TOP2A and KIF14), therefore were recognized as MK167+ CP. In contrast, five clusters, including C0, C1, C2, C5, and C6, expressed the markers of cone precursors but expressed low level of MKI67, suggesting that these cells were less proliferative, therefore were recognized as CP-like (Figs. 1D, S2D). C16 was interpreted as mature cone-like cells with strong expression of PDC, ARR3 and GNGT2, while C13 was rod-like cells with strong expression of PDC, RHO, and PDE6A (Figs. 1D, S2D).

Except for photoreceptor-related markers, we also detected other marker genes. We found that C15 specially and highly expressed CD68, HLA-DPA1, HLA-DPB1 and CLU [11], indicating that C15 had immune properties and thus recognized as glial cells (Figs. 1D, S2D). C12, highly expressed ACTA2, VIM, and FGF9 [11, 30], was recognized as cancer-associated fibroblasts (CAFs) (Figs. 1D, S2D). Notably, we found that the RB1 gene was only expressed in glial cells and CAFs (Fig. S2E), indicating that glial cells and CAFs, as components of TME, were distinctly different from other cells. C11 did not express cell-specific markers, but the enrichment analysis of differentially expressed genes (DEGs) showed that this cluster mainly performed neural-related functions (Fig. S2D). Therefore, we named C11 as neural cells. Besides, C4 and C14 expressed markers from multiple ribosomal genes and therefore were excluded from further analysis. The details of the identified markers and cell numbers of individual cell types were listed in Table S4.

Principal component analysis (PCA) showed that CP-like and MKI67+ CP cells were clustered closer but separated from the microenvironment related glial cells and CAFs. As expected, subtypes of the same cell-type clustered together (Fig. 1C). By comparing the cell composition in RB, we found that most of the cells were CP-like (~68%), followed by MKI67+ CP cells (~28%) (Fig. 1E). The cell types in RB were clearly different from those in normal human retina, where rod photoreceptors and BCs formed most cells [25, 31, 32]. Notably, the other retinal cell types (such as RPE, PEC, RGC, AC, BC and HC) related markers (such as SOX2, MITF, POU4F2, PROX1, VSX2, and PAX) were not expressed in all the clusters (Fig. S2D). In contrast, 96% of the cells contained characteristics of CP-like cells, suggesting that RB may originate from CP rather than the other cell types in retina, consistent with previous findings [22, 29]. In summary, we presented a detailed characterization map of cell types and comprehensive transcriptome landscape of human RB in a single-cell resolution.

### Malignant cells defined by copy number variations in RB

**Fig. 2:**
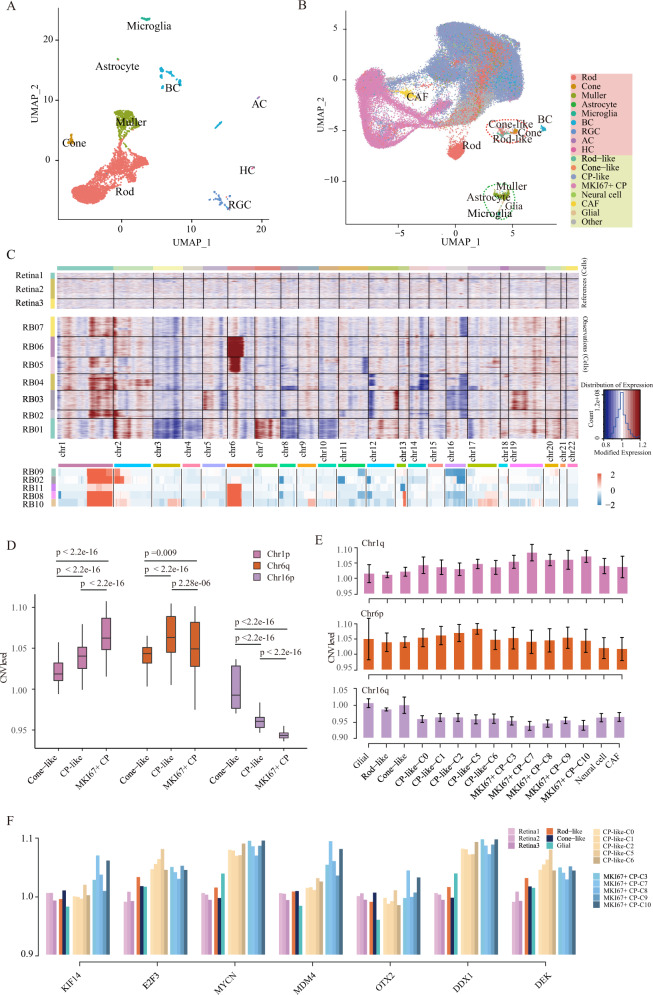
Clonal heterogeneity defines RB malignant cells. **A** U-MAP visualization of about 4000 retinal cells colored by cell types. **B** U-MAP visualization of RB and retinal cell colored by cell types after integration through CCA. Red background in the right panel represents normal retinal cell types, and yellow represents tumor cell types. **C** Chromosomal landscape of inferred CNVs based on scRNA-seq and WES. The x-axis shows the chromosome region, the y-axis represents the samples, and color represents the CNV level. The top is the copy number of the normal retina (Reference), the middle is the copy number of the RB sample (Observations), and the bottom is the inferred CNV from WES. **D** Box plot for CNV level of cone-like, CP-like, MKI67+ CP on Chr1p, Chr6q, and Chr16p, all cells in each cluster are averaged on the same segment. **E** CNV level of RB cell types on Chr1p, Chr6q, and Chr16p. **F** The box plot shows CNV level of KIF14, E2F3, MYCN, OTX2, DDX1, and DEK in cell types of retina and RB.

RB patients with surgery were in the late stage and the tumor samples lacked normal or precancerous cells. We used a single-cell profile of a normal human retina as normal control for further analysis (downloaded from EBI: E-MTAB-7316) [25]. After processing and filtering, 4657 single cells were used to define 16 cell clusters, including 9 major retinal cell types, which was consistent with the original report (Figs. 2A, S3A–C, Methods). By integrating the single-cell expression profiles of normal retina and RB, we found that the similar cell types in RB and retina could be well integrated together (Fig. 2B). For example, the cone-like cells presented in RB could aggregate with cone cells of the normal retina, and RB glial cells and retinal microglia cells also clustered together (Fig. 2B). Our analysis indicated that small percentage of cells in RB retained the characteristics of normal retinal photoreceptor cells, while most cells were unique to tumor and more likely to be tumor malignant cells.

To define the malignant status of cells, we inferred CNVs for each cell by averaging relative expression levels across intervals of the genome (Methods) [14, 33]. Based on inferred CNV profiling from the retina and RB scRNA-seq datasets, we found different types of CNVs in the RB tumor samples (Fig. 2C), including gain of chromosome (Chr) 6p (Samples: RB05 and RB06), gain of Chr1q and loss of Chr16q (RB02, RB03, RB04, and RB07), and gain of Chr7 and losses of Chr3, 8, 10 (RB01). The most frequent of amplified regions such as Chr1q and Chr6p, and the most frequent of deleted regions such as Chr16q were consistent with previous findings [34]. To confirm the accuracy of our inferred CNVs, we also sequenced five RB samples with match blood samples by whole-exome sequencing (WES), which showed similar CNV patterns in RB (Fig. 2C; Methods). For example, comparative analysis of CNV profiles of RB02 by scRNA-seq and WES showed the same changes, with common amplification in Chr1, 2 and deletion in Chr8, 11, 12, and 16.

Based on the inferred CNVs, we found that the CP-like and MKI67+ CP cells were malignant cells, which were characterized by either the amplified copies in Chr1q or Chr6p or deleted copies in Chr16q (Fig. S4A). On the other hand, glial, cone-like and rod-like cells tended to be normal cells, which had no copy number changes in these specific regions (Fig. S4A). In addition to the use of normal retina as a reference, we also used immune cells (here glial) as an alternative reference to infer malignant cells and found the similar changing pattern in copy number (Fig. S4B).

Comparative analysis of CNV levels further revealed different degrees of malignancy among cell types or even subtypes. For example, the highest gain and loss in Chr1q and Chr16q regions found in MKI67+ CP, followed by CP-like and cone-like (two-sided *p* values < 0.01 for all the pairwise comparison, paired *t* test, Fig. 2D). Among the subtypes of MKI67+ CP, C7 and C10 exhibited remarkably higher CNV gains on Chr1q and CNV losses on Chr16p than other subtypes (Fig. 2E). Interestingly, previous studies reported that some oncogenes, such as KIF14 and MDM4 in Chr1q region, had copy number gains during the transition from retinoma into RB [2, 35]. We also found that these genes were amplified in MKI67+ CP, particularly in the C7 and C10 subtypes (Fig. 2F). Altogether, our results suggested that MKI67+ CP cells, particularly the C7 and C10 subtypes, were more malignant in RB.

### Functional diversity and transition among RB malignant cell types

**Fig. 3:**
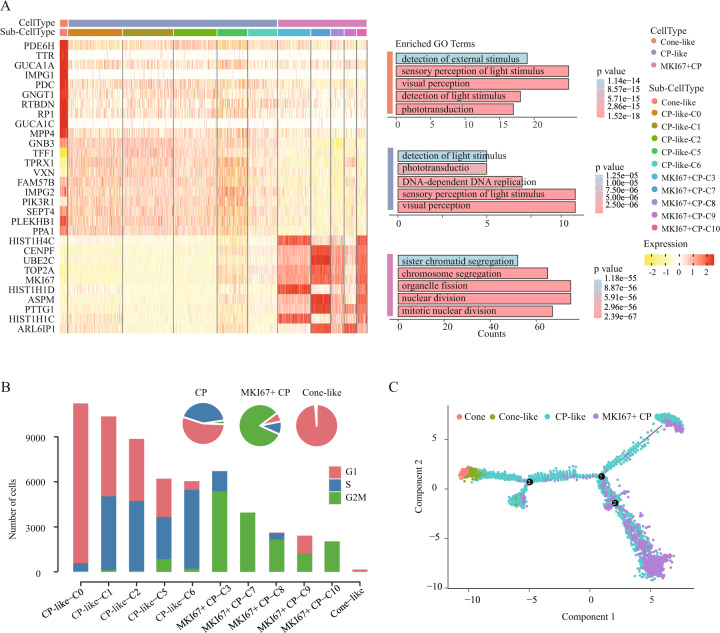
Cellular diversity in RB malignant cells. **A** Heatmap of significantly differentially expressed genes (left) and functional enrichment (right) of the major cell types. **B** Cell cycle status of cone-related clusters. **C** Developmental pseudo-time of cone-related clusters, colored according to cell types.

To further understand the functional relevance of cell types in RB, we compared their functional differences. To do this, we identified 833, 327, and 330 DEGs in cone-like, CP-like and MKI67+ CP cells, respectively. The enrichment analysis showed that the upregulated genes in cone-like cells were significantly enriched for several visual-related functions, such as phototransduction, visual perception, detection of light stimulus, suggesting that cone-like cells maintain the functions of normal cone cells (Figs. 3A, S5A, D). The upregulated genes in MKI67+ CP included some oncogenes, such as MKI67, KIF14, UBE2C, PTTG1, and CDC20, and were mainly enriched in cell cycle-related functions, such as nuclear division and cell cycle G2/M phase transition, suggesting a high cell proliferation capability (Figs. 3A, S5C, F). Among the subtypes of MKI67+ CP, they also existed some functional differences, such as nuclear division and chromosome segregation exclusively found in C7 and C10, indicating their stronger proliferation (Fig. S5G). Notably, we found that CP-like cells retained some characteristics of cone-like cells, such as response to light stimulus, while being enriched in some metabolic-related processes, such as hexose metabolic process and glucose metabolic process (Figs. 3A, S5B, E). These results showed that the CP-like cells with the highest proportion in RB might act as the intermediate cells in the malignant stage, therefore transforming to MKI67+ CP. During transformation of this process, the photoreceptor function decreased, and the cell proliferation function were strengthened.

Notably, although proliferation signature of G2M phase was observed in RB [8], the cell cycle states for specific cell types were unclear. Here, we conducted cell cycle analysis (Methods) and found that almost all the cone-like cells were associated with G1 phases, illustrating that they were stagnant in division without proliferation and differentiation potential. Compared with cone-like cells, CP-like cells contained 50% in the G1 phase, 45% in the S phase and 5% in the G2M phase, indicating their proliferation potential. Moreover, 75% of the MKI67+ CP cells were found in the G2M phase, particularly C7 and C10 are almost 100%, indicating their strong proliferation potential (Fig. 3B). Consistent with the results of functional enrichment analysis, such an analysis further confirmed that the CP-like cells might be intermediate cells, and the transformed MKI67+ CP cells were in high proliferation state and were highly malignancy. Next, we explored the transition among cone, cone-like, CP-like and MKI67+ CP cells by performing trajectory analysis (Methods). We recapitulated the tumor differentiation and growth process and observed that the CP-like cells are mainly enriched in the intermediate cell state, while the other cell types are mainly enriched in the end of trajectory branches (Figs. 3C, S6A, B). Of note, the normal or tending to normal cells (cone and cone-like) and the malignant cells (MKI67+ CP) were distributed towards the opposite directions, suggesting CP-like cells may differentiate into MKI67+ CP cells, or into a small number of cone-like cells. Notably, among the subtypes of MKI67+ CP, C7, and C10 were at the end of multiple branches (Fig. S6C). In summary, MKI67+ CP-C7 and -C10 had the strongest proliferation capability, which might be the potential targets for future therapies.

### Tumor immune microenvironment in RB

**Fig. 4:**
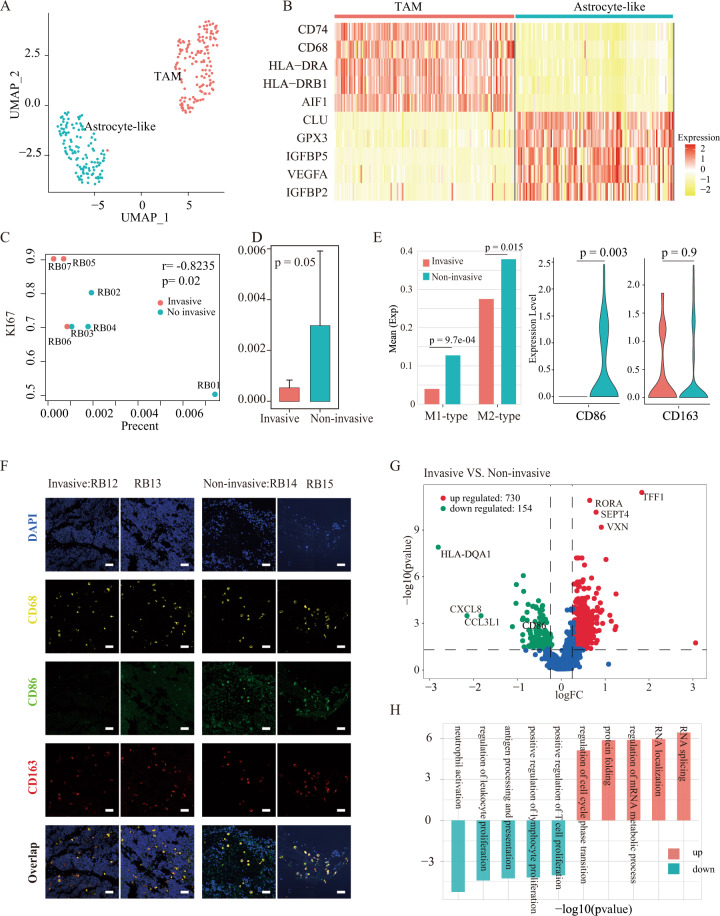
Regulation of microenvironment in human RB. **A** u-MAP visualization of the cells in the C15 cluster (Glial cells). **B** Heatmap of 10 significantly differentially expressed genes between macrophages and astrocytes. **C** Scatter plot of the proportion of TAMs between invasive (orange) and non-invasive (green) samples, classified by clinical pathological information. **D** Box plot of the proportion of TAMs in different samples; **E** Box plot of the average expression levels of M1 and M2-type related genes in the invasive and non-invasive groups (left); Violin plot of CD86 and CD163 expression levels in the invasive and non-invasive groups (right). **F** Expression of TAMs cellular markers CD68, M1-type CD86, and M2-type CD163 in FFPE sections of RB by immunohistochemistry. The left is invasion samples, the right is non-invasion samples (original magnification, ×50). **G** Volcano plot for differentially expressed genes in the invasive and non-invasive samples. **H** Functional differences of TAMs between the invasive and non-invasive samples.

Given the immune-related marker gene and functional relevance of the C15 cluster (glial cells) (Fig. S7A, B), we conducted unsupervised dimensionality reduction and clustering to identify immune cell subtypes, including C15_C0 and C15_C1 (Fig. S7C). The C15_C0 cluster mainly expressed the macrophage cells marker gene (CD68, CD74) and C15_C1 mainly expressed the astrocyte cells marker genes (CLU, PAX2) (Fig. S7D), illustrating that the immune microenvironment in RB tumors mainly consisted of tumor-associated macrophages (TAMs) and astrocyte-like cells (Fig. 4A), consistent with previous findings [11]. After identifying highly expressed genes in TAMs and astrocyte-like (Fig. 4B), enrichment analysis further showed that TAMs were mainly responsible for regulation of cell adhesion, lymphocyte activation, and antigen processing and presentation, while astrocyte-like were mainly responsible for metabolic process, endopeptidase activity, and response to hypoxia and oxygen (Fig. S8A, B).

Interestingly, the proportion of TAMs had significantly negative correlation with the KI-67 index of the patients (*r* = −0.82, *p* = 0.02, Pearson correlation), given that the KI-67 index, a clinical indicator of tumor proliferation, is often used to reflect the malignant degree of tumor, with high index representing high malignant capability (Fig. 4C). Furthermore, based on patients’ clinical pathological information (Table S1), we found that the proportion of TAMs in the non-invasive group was 11.83-fold higher than that in the invasive group (*p* = 0.05, Wilcoxon-test) (Fig. 4D). The decreased number of TAMs in the invasive RB group suggested a potential immune depletion. In addition, considering published signature gene lists for M1-type (classically activated macrophage) and M2-type (alternatively activated macrophage) (Table S5), we found that M1-type related genes were the lowest expression in the invasion group, especially CD86, one typical M1 marker gene, was not expressed in the invasive group (Fig. 4E), where the presence of M1-type macrophages may help to secret pro-inflammatory cytokines and chemokines, present antigens and participate in the positive immune response [36]. In contrast, a marker of M2-type (alternatively activated macrophage), CD163, was expressed in both groups but no difference (Fig. 4E, *p* = 0.9, Wilcox test). These findings were also confirmed by our immunohistochemistry (Fig. 4F). Altogether, these results suggested that during the invasion process, M1-type macrophages may be regressed or transformed into M2-type macrophages. Differentially expressed gene analysis for TAMs identified 720 upregulated and 154 downregulated genes in the invasive group compared to the non-invasive group (Fig. 4G). Functional enrichment analysis further revealed that downregulated genes were involved in immune-related functions, such as lymphocyte proliferation, leukocyte cell–cell adhesion, and neutrophil mediated immunity, indicating immune function loss during the invasion process (Fig. 4H).

We also observed that the number of astrocyte-likes cells had negative correlation with KI-67 index with marginal significance (*p* = 0.06, *r* = −0.72, Pearson correlation) and the average proportion of astrocyte-like cells in the invasive group were only 48% of that in the non-invasive group (Fig. S9A, B), showing that the number of astrocyte-like cells also decreased during the invasion process. By comparing with the expression profiles of the non-invasive, we identified 418 upregulated genes and 133 downregulated genes in the invasive group (Fig. S9C). We found that downregulated genes were mainly enriched in response to reactive oxygen species, response to hydrogen peroxide, negative regulation of hydrogen peroxide-induced cell death and response to calcium ion (Fig. S9D). These functions were found to play important roles in cancer [37–41], for instance, hydrogen peroxide could inhibit the growth of cancer cells, that were used as potential therapy targets for several type of cancer [42, 43]. In summary, our analysis suggested that TAMs and astrocyte-like cells created an immunosuppressive environment during the invasion process in RB.

### Inference of intercellular interactions in RB

**Fig. 5:**
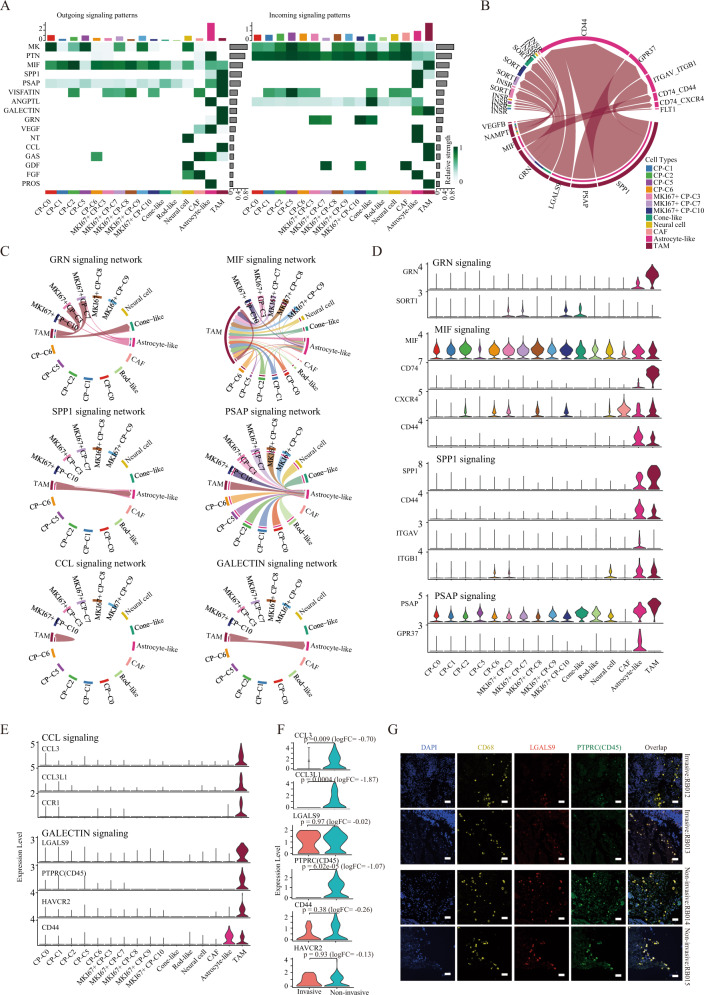
Intercellular and molecular interactions in RB tumors. **A** The heatmap shows the relative strength of the outgoing signaling pathway (left) and the incoming signaling pathway (right) across 16 cell types. The color represents the relative signaling strength. The top colored bar plot shows the total signaling strength of a cell-type by summarizing all signaling pathways displayed in the heatmap. The right gray bar plot shows the total signaling strength of a signaling pathway by summarizing all cell types displayed in the heatmap. **B** This chord diagram shows specific connections between TAMs ligands and other cell types receptors. The color represents different cell types, and the thickness of the line represents the strength of the connection. **C** This chord diagram shows TAMs-mediated signaling pathway networks, including GRN, MIF, SPP1, PSAP, CCL, and GALECTIN signaling networks. **D** Violin plot shows the expression of the GRN, MIF, SPP1 and PSAP signaling pathway related ligands and receptors in 16 cell types. **E** Violin plot shows the expression of the CCL and GALECTIN signaling pathways related genes across 16 cell types. **F** Violin plot shows the expression of the CCL and GALECTIN signaling pathways related genes in the invasive and non-invasive groups. **G** Expression of the CD68 (TAM marker gene), GALECTIN signal related ligand LGALS9 and receptor PTPRC (CD45) in FFPE sections of invasive and non-invasive RB samples (original magnification, ×50).

Understanding interactions between the non-neoplastic and neoplastic cells in cancer is important to comprehend the mechanism of cancer progression. We next inferred intercellular communications between different cell types using CellChat [44] (Methods). A total of 16 significant signaling pathways were predicted among the 16 cell types (Fig. 5A), where TAMs received the strongest incoming signals and astrocyte-like cells generated the strongest outgoing signals (Fig. 5A). Notably, many of the high-scoring interactions such as MIF-CD74, SPP1-CD44 were observed between TAMs and astrocyte-like (Fig. 5B).

Previous studies showed the importance of macrophage-derived Granulin (GRN) in driving resistance to immune checkpoint inhibition in metastatic cancer [45]. We found that TAMs cooperated with astrocyte-like cells to regulate MKI67+ CP-C3, C7, and C10 cells through the GRN-SORT1 pair, in which the ligand GRN was mainly expressed in TAMs, and the receptor SORT1 was expressed in MKI67+ CP-C3, C7, and C10 (Fig. 5C, D). Previous finding showed that MIF was overexpressed in these malignancies in humans, and contributes to the deregulation of angiogenesis, and metastasis [46], we found that almost all malignant cells regulated TAM cells through the ligand MIF and the receptors CD74 (Fig. 5C, D). Another example was the SPP1 and PSAP signals. The former has been showed to promote the progress of cancer through modulation of vascular endothelial growth factor expression [47–49] and the latter has been showed to be essential for migration of astrocytes [50]. We found that the SPP1 signal was mainly involved in the interaction between TAMs and astrocyte-like cells through SPP1-CD44, -ITGAV, and -ITGB1 pairs, and PSAP signal involved that TAMs regulated astrocyte-like cells through PSAP-GPR37 pair (Fig. 5C, D).

Besides intercellular communications, TAMs, serving as the sender and receiver of CCL signal and GALECTIN signal, also exhibited intra-cellular communications through CCL3-CCL3L1 and -CCR1 pairs as well as LGALS9-CD45, -HAVCR2, and -CD44 pairs, respectively (Fig. 5C, E). Notably, an important chemokine implicated in both immune surveillance and tolerance [51], CCL3 (*p* = 0.009, logFC = −0.70) and CCL3L1 (*p* = 0.0004, logFC = −1.87) were downregulated in the TAM of the invasion group (Fig. 5F), indicating that the CCL signal was weakened during the invasion process. LGALS9, CD45, HAVCR2, and CD44 also tended to be downregulated in the invasive group (Fig. 5F) and meanwhile our immunohistochemistry confirmed their reduced expression (Fig. 5G), suggesting that this signal was inactivated during the invasion, consistent with previous findings of an anti-proliferative (metastatic) effect of LGALS9 (-HAVCR2) on cancer cells [52]. To sum, these results highlighted the important pathways mediated by TAMs in RB, particularly for the CCL and GALECTIN signals, which were related to the self-regulation of TAMs and cancer invasion.

## Discussion

The current treatments of RB are gradually shifting towards more advanced tailored therapies that could preserve useful vision and reduce treatment-related risks [53, 54]. Therefore, it is important to understand how the intratumoral heterogeneity and microenvironment are related to the occurrence and malignant degrees of RB, which will facilitate development of new treatment strategy of RB. Recently, two RB single-cell studies [22, 23] discussed the heterogeneity of RB tumors. Because these two studies included only two samples, with a relatively small number of cells (8086/14,739), they did not identify many rare cell types, such as CAF, TAM, and astrocyte-like, which had been confirmed to exist in RB [11]. In addition, we still lack understanding how microenvironment alteration are related to the invasion RB.

Here, we showed that there was strong clonal heterogeneity of malignant cells among seven RB samples. In addition to common Chr1p and Chr6q regions copy number amplification and Chr16p copy number deletion, we found some novel CNVs such as copy number amplification in chr2p and Chr7q, and deletion in Chr8 and Chr10 regions. Although CP-like and MKI67+ CP accounting for major malignant cells, there were still large CNV differences between cell subtypes. Two subtypes (C7 and C10) of MKI67+ CP cells had significantly larger copy number changes, indicating higher degree of malignancy. Notably, the heterogeneity of RB not only reflected in the difference of CNV, but also in difference of cellular functions. MKI67+ CP cells were mainly associated with cell proliferation function, of which the C7 and C10 subtype cells had stronger proliferation ability. In addition, these two subtypes were at the end of differentiation by trajectory analysis. Our finding may give an inspiration for the treatment of RB by inhibiting the proliferation of MKI67+ CP cells, particularly the C7 and C10 subtypes.

A better understanding of TME in RB and the potential for immunotherapy may lead to novel therapeutic strategies. We found that TME in RB was mainly composed of TAMs, astrocyte-like and CAF cells. Our study showed a few lymphocytes, particularly T cells, in RB. This was consistent with recent studies also showing the absence of CD8+ cells, including T cells, B cells, in RB [55]. These results suggested that RB has relatively poor immunogenicity compared with most of tumors, which may contribute to the poor efficacy of immunotherapy in RB. Besides, we found that during the invasion process, the proportion of TAMs and astrocyte-like decreased, M1-type macrophage was lost, and the immune functions of TAMs and anti-cancer functions of astrocyte-like were also depressed, which may reflect an immunosuppressive environment. The number of TAMs may indicate the invasion of RB patients. By calculating the information interaction between cells, we discovered that GALECTIN and CCL, two important pathways that mediated immunosuppression, were inhibited during the invasion process, indicating its anti-invasion effect and its potential as an immunotherapy target. In addition, previous studies have reported that TAMs were related to tumor vascularization [56, 57], and expression of GRN by TAMs was able to increase their angiogenic potential in breast cancer [58]. Interestingly, we also found that GRN was expressed in TAMs in RB (Fig. S11A), and the expression of GRN was positively correlated with the expression of angiogenesis-related genes (*p* = 0.0002, *r* = 0.313, Pearson correlation, Table S5, Fig. S11B), indicating the ability of TAM to promote angiogenesis through GRN expression in RB.

In addition to TAMs and astrocyte-like cells, we found that CAFs were characterized by highly expressed cell proliferation-related genes, such as PLK2, ATF3, SNHG12, HJURP, E2F8, and EXO1 (Fig. S10A), and significantly enriched by many cell proliferation and cancer-related functions in CAFs, such as cell cycle, DNA replication, DNA damage and p53 signaling pathway (Fig. S10B, C). Further analysis, we found that CAFs interacted with astrocyte-like and TAMs through FGF and GAS signals (Fig. S12A). Previous studies showed that CAFs promoted tumor progression through the activation of FGF signaling in a variety of cancers, including colon cancer [59], skin squamous cell carcinomas [60], lung adenocarcinoma [61] and ovarian cance [62]. Here, we found that FDF9 from CAFs and neural cells was the primary ligand for FGF signaling, which bound to the receptor PGFR1 secreted by astrocyte-like cells (Fig. S12B). GAS signal contained GAS6-AXL pair, which was mainly mediated by CAFs and TAMs (Fig. S12A, B). In the TME, CAFs express Gas6 [63] and the Gas6/Axl signaling pathway has been implicated in the promotion of tumor cell proliferation, survival, migration, invasion, angiogenesis, and immune evasion [64]. These results indicated that CAFs might play a role in promoting tumor proliferation.

In summary, our data provided a valuable resource for deciphering the comprehensive gene expression landscape of heterogeneous tumor and immune cell types in RB, which will facilitate the understanding of the mechanism of RB progression and give clues to future treatment of RB.

## Materials and methods

### Human RB samples

Eyes of patients with clinical diagnosis of RB were enucleated through surgery at the department of Sun Yat-sen University Eye Hospital. All experiments involving patients had signed the consent forms and the proposed studies were approved by Ethics Committee (2021KYPJ065). Immunohistochemistry sections were obtained from primary tumor tissues and examined by professional pathologists. Histological characteristics and Ki-67 proliferation status were evaluated after surgery. The peripheral blood of the patient was used for targeted exon sequencing of 126 common ocular genetic pathogenic genes, and no pathogenic mutations in the RB1 gene were detected. Age, gender, RB1 germline mutation status, histological pathology and Ki-67 index of patients involved were listed (Table S1).

### Single-cell RNA sequencing

We collected fresh RB tumor tissues from the enucleated eyeballs. Single cells of RB tumor tissues were isolated by using Neuronal Isolation Enzyme (with papain) (Thermo Fisher, #88285).The libraries were constructed using the Chromium platform and Chromium Single-Cell 3′v2 chemistry. Briefly, cellular suspensions were loaded on a Chromium Single-Cell Instrument (10x Genomics, Pleasanton, CA) for GEMs Generation and Barcoding. Single-cell RNA-Seq libraries were prepared using the Chromium Single-Cell Library Kit (10x Genomics, Pleasanton, CA). Single-cell libraries were sequenced in 150 nt paired-end configuration using an Illumina HiSeqX10 and Novaseq.

### Whole-exome sequencing

Genomic DNA was extracted from five RB samples (tissue and blood) using Qiagen DNAeasy kits. Adaptor-ligated libraries were constructed using Paired-End DNA kits. The exome capture was performed by using SureSelect Human All Exon, followed by 150 nt paired-end whole-exome sequencing on Illumina NovaSeq platform.

### 10X Genomics cell ranger pipeline

Illumina BCL files were converted to fastq files by using 10X Genomics Cell Ranger pipeline mkfastq function (v 2.1.0; https://support.10xgenomics.com/). Next, cell ranger count function was used to generate Gene-Barcode matrices from the fastq files, which uses the STAR algorithm to map high-quality reads to the human reference genome (GRCh38), followed by UMI counting. The number of estimated cells, average reads and median genes in each sample was listed (Table S2).

### Single-cell data processing

Gene-Barcode matrix of each sample was imported into Seurat package (v3.2.0; https://github.com/satijalab/seurat). We calculated the expressed cell number in each gene, the number of expressed genes in each cell, and the ratio of mitochondrial genes, red blood cell genes, ribosomal genes, which was used as quality control to remove outlier genes and cells. Genes expressed in a minimum of ten cells and cells with 1000–4000 detected genes, expressing <5% mitochondrial genes, expressing <10% red blood cell and expressing <40% ribosomal genes were retained. After filtering, the number of cells and genes in each sample was listed in Table S2. Next, data was normalized using NormalizeData() and 2000 high variable genes were identified with the FindVariableGenes(). Data from multiple samples were merged using the FindIntegrationAnchors() and IntegrateData() function with the first 20 CC dimensions. The integrated dataset was scaled and reduced into a lower dimensional space using principal components analysis (PCA). The top 30 principal components were used as inputs for graph-based cell clustering at a resolution value of 0.6. Clustering results were visualized using Uniform Manifold Approximation and Projection (UMAP) with Seurat functions RunUMAP.

### Identification of RB cell types

Marker genes of each cluster were identified by using FindAllMarkers() function in Seurat, which compared each cluster to all others combined using the Wilcoxon method. Marker genes expressed in a minimum of 25% of cells and at a minimum log fold change threshold of 0.25 were retained (Supplementary File 1). Due to the lack of known marker genes for RB cell types, we manually collected a set of well-established marker genes that covering all known major cell types in the retina (Table S3). We assessed the putative identity of each cell through the expression of the known marker genes in each cluster.

### Copy number variation (CNV) analysis based on scRNA-seq

CNV was estimated by using the R package inferCNV (https://github.com/broadinstitute/inferCNV; v1.6.0), which sort the genes according to their chromosomal location and apply a moving average to the relative expression values. Here, to infer the CNVs of RB cells (69820 cells), we use 3 retinal samples (4657 cells) as a reference, the count matrix as input, and calculate the copy number changes of all cells using the infercnv::run() function with setting parameters cutoff = 0.1, denoise = TRUE, cluster_by_groups = TRUE and HMM = TRUE. For the grouping information of cells, we use two types, one is by sample, the other is by cell type. Notably, we also used glial cells as a reference to calculate the CNV of other cells in RB with the same parameters as above.

### Correlation analysis

The mean expression levels of all cells in each sample were calculated, which is used to calculate the Pearson’s correlation coefficient between samples. Similar strategy is used to evaluate the correlation between bulk RNA-seq and single-cell RNA-seq expression profiles.

### Go function enrichment and KEGG pathway analysis

Enrichment analysis for detected significant DEGs was performed using clusterProfiler package [65], which can analyze and visualize functional profiles (GO and KEGG).

### Cell cycle prediction

Cell cycle was predicted using CellCycleScoring() of Seurat. In brief, the normalized scores of S phase and G2/M phase of each cell was calculated by using the average expression levels of 43 S phase marker genes and 54 G2/M phase marker genes (Seurat package provided), and then assigns each cell to the phase with the highest score (if both scores are negative, the cell is assigned to G1 phase).

### Pseudo-time analysis

Monocle [66] (v2) was used to perform pseudo-time analysis to explore the development trajectory which introduced the strategy of ordering single cells in pseudo-time. After identifying the cell types by Seurat, we selected cone-related cell types, including cone (101 cells), cone-like (157 cells), CP-like (42692 cells) and MKI67+ CP (17731 cells) for trajectory analysis. Due to the excessive number of CP-like and MKI67+ CP cells, we randomly selected 10% of the CP-like cells and 5% of the MKI67+ CP cells for testing. Here, the count matrix was used as input. We first screened the genes that expressed in at least 500 cells, and identifed differentially expressed genes (DEGs, qval <0.01) using the differentialGeneTest(). These DEGs were chose to define a cell’s progress. Next, we reduced the space down to one with two dimensions to easily visualize and interpret while Monocle is ordering the cells using reduceDimension() function with the following parameters: max_components = 2, method = “DDRTree”. Finally, the orderCells() was used to sort the cells, and the plot_cell_trajectory() function was used for visualization. Since some cells were randomly selected, we repeated this process 5 times to check whether the trajectory has changed (Fig. S6A, B), and found that the trajectory was basically similar.

### Cell–cell interaction analysis

CellChat (V1.1.2) was used to infer the RB cell–cell communications and significant pathways by integrating gene expression with prior knowledge of the interactions between signaling ligands, receptors and their cofactors. To identify potential cell–cell interactions that were perturbed or induced in RB samples, we focused on differentially expressed ligands and receptors (*P* < 0.05) in 16 cell types, including TAM, CP-like, MKI67-CP, CAF, etc. Briefly, we followed the official workflow and loaded normalized data (19956 genes and 62747 cells) into createCellChat() to create a CellChat objects. Next, we loaded CellChatDB.human ligand–receptor database and used the “Secreted Signaling” pathways for cell–cell communication analysis. Then, identifyOverExpressedGenes() and identifyOverExpressedInteractions() with default parameters were used to identify overexpressed signaling genes and ligand–receptor interactions (pairs) associated with each cell type; ComputeCommunProb() and computeCommunProbPathway() functions with default parameters were used to identify putative interaction pairs and pathways. Finally, netAnalysis_computeCentrality() function was applied on the netP data to determine the senders and receivers, and netVisual() function was used to visualize communication network associated with both signaling pathway and individual L-R pairs.

### Whole-exome sequencing analysis

Quality control was performed using Fastp software to filter low-quality reads [67]. Read pairs were aligned to the reference human genome hg38 using the Burrows-Wheeler Aligner (BWA) [68]. Bam files were duplicate-marked using Picard, and base quality recalibration was performed using Genome Analysis Toolkit toolbox (GATK4.0.9) [69]. Next, copy number variants were identified using CNVkit [70]. Finally, we summarized segment calls to gene levels copy number by GISTIC2.0 [71], which identifies regions of the genome that are significantly amplified or deleted across a set of samples. The gene copy number matrix was listed in Supplementary File 2.

### Immunohistochemistry (IHC)

For immunological experiment, the whole RB eyeballs were embedded in paraffin, and serial 4-μm-thick sections were cut by transecting near the optic nerve. The sections were stained with the following primary antibodies: CD68 (ab955; Abcam) to detect TAMs, CD86(14-0862; Invitrogen), CD163(ab182422; Abcam), PTPRC (CD45) (14-0451; Invitrogen) and LGALS9 (ab69630; Abcam). Alexa Fluor secondary antibodies (Invitrogen) were used for detection of primary antibodies. DAPI was used to label nuclei. Imaging was performed with a Zeiss LSM 880 confocal microscope at ×20 magnifications. Images were visualized with Zeiss Zen software (blue edition; v.2.5).

### Public datasets

We downloaded the fastq files of retinal single-cell RNA-seq at ArrayExpress under the accession number E-MTAB-7316 (http://www.ebi.ac.uk/arrayexpress/experiments/E-MTAB-7316) [25]. Single cells from three independent neural retina samples were captured in five batches using the 10X Chromium system. FASTQ files were mapped to the human genome (GRCh38) and all libraries were aggregated to the same effective sequencing depth with the Cell Ranger pipeline (cell ranger aggr function). We initially obtained 14,829 cells. After adopting the same quality control as RB, we obtained 4,657 high-quality cells. Next, PCA was carried out, and the top 30 PCs were retained. Clustering was performed with the clustering resolution set to 0.6, and 14 cell clusters were identified. 9 cell types were determined by known marker genes’ expression. For the integration analysis of retinal dataset and RB dataset, we also used FindIntegrationAnchors() function to integrate in Seurat. All parameters were the same as those used in the above analysis of RB. Besides, we downloaded mRNA profiles of RB samples [7] and para-tumor [3] from GSE125903 [72], which were generated by RNA sequencing.

## Supplementary information

Supplementary Information

Reproducibility checklist

## Data Availability

The scRNA-seq data were deposited in the GEO database (accession number: GSE168434).

## Code availability

Processed and analyzed data and partially analysis scripts generated during this study are available at GitHub https://github.com/Wucheng-tech/Retinoblastoma.git (master).
