# Supplementary material for: Single-cell characterization of malignant phenotypes and microenvironment alteration in retinoblastoma
Source: Cell Death Dis. 2022 May 6;13(5):438. doi: 10.1038/s41419-022-04904-8 (PMC9076657; doi:10.1038/s41419-022-04904-8)
Supplement: Supplementary file 1 — Supplementary Information [file 41419_2022_4904_MOESM1_ESM.docx]

**Supplementary Information**

Single-Cell Characterization of Malignant Phenotypes and Microenvironment Alteration in Retinoblastoma

**This file includes:**

**Supplementary Fig. S1-12**

**Supplementary Tables 1-5.**


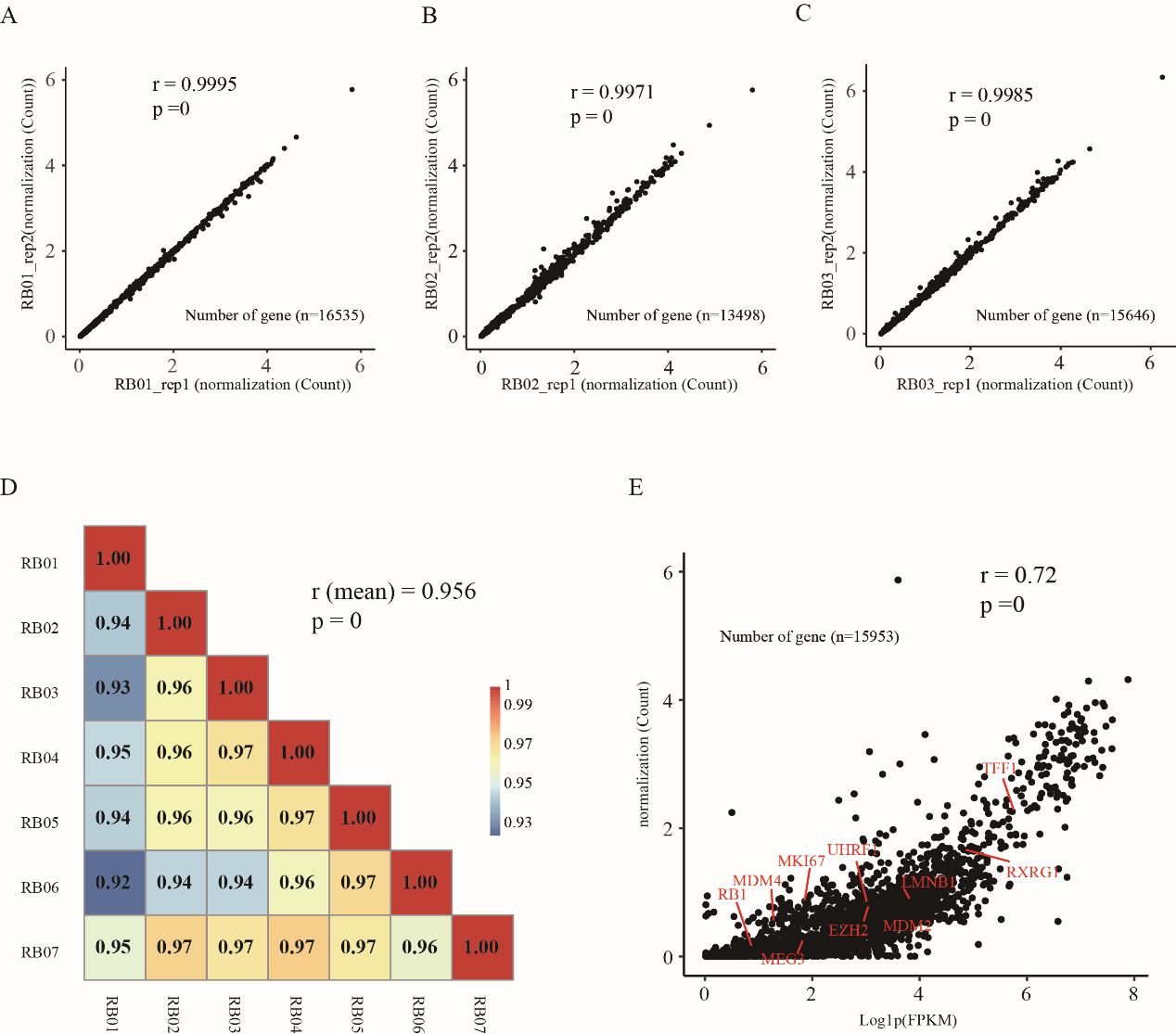


**Figure S1.** Pearson correlation analysis. (A, B, C) The Pearson correlation between single cell RNA expressions profiles in repeated sequencing samples, where a point represents a gene; (A) RB01_rep1 VS. RB01_rep2; (B) RB02_rep1 VS. RB02_rep2; (C) RB03_rep1 VS. RB03_rep2. (D) The Pearson correlation between single cell RNA expressions profiles in different samples; Red is a strong correlation and blue is relatively weak. (E) The Pearson correlation between the single-cell and the bulk expression profiles in RB. The X axis represents the average expression level of each gene in the single cell expression profile, and the Y axis represents the expression level of each gene in the bulk expression profile.


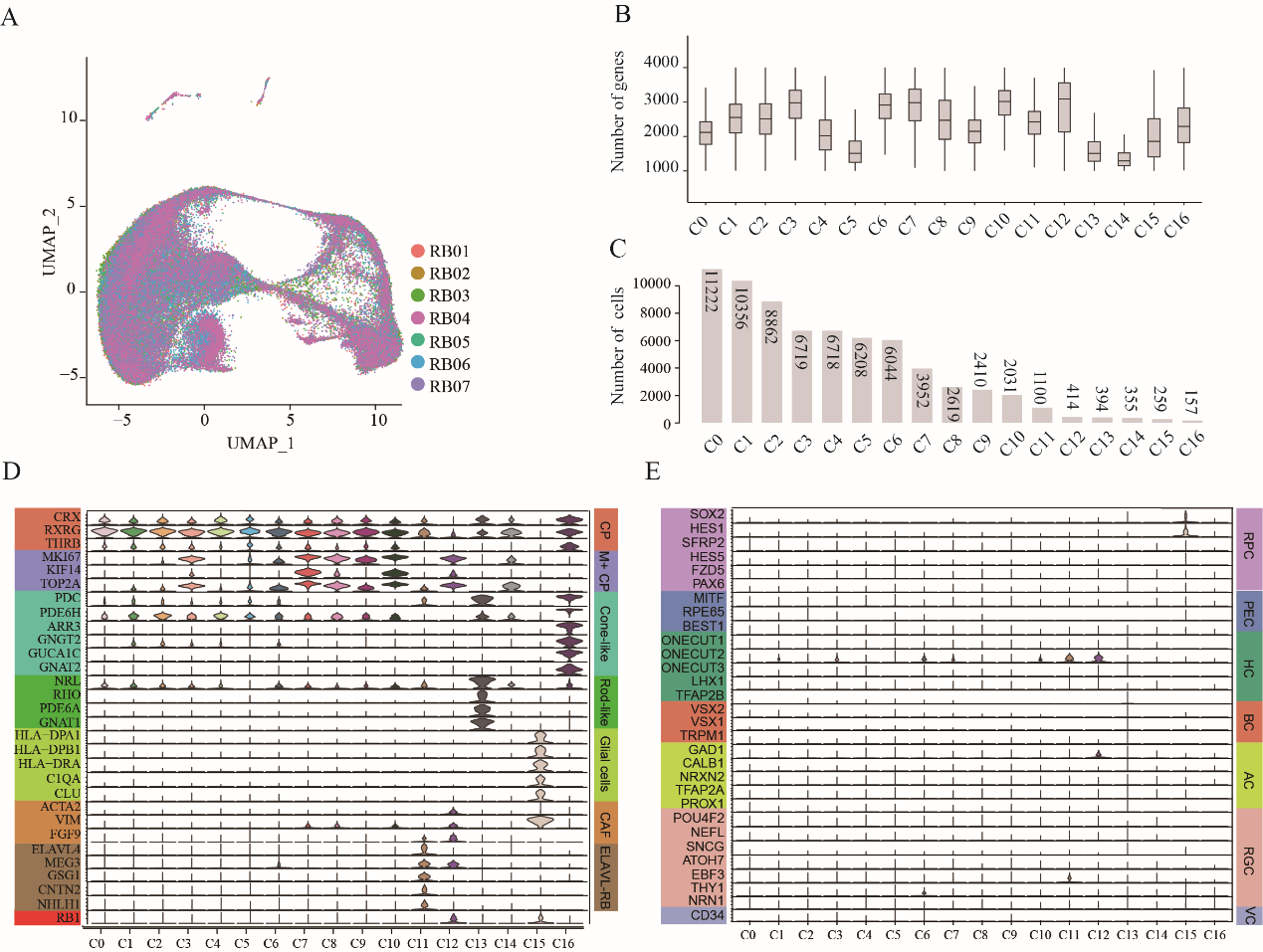


**Figure S2.** (A) U-MAP visualization of about 70000 cells using CCA in RB. (B) The box plot shows the number of genes in each cluster. (C) The histogram shows the number of cells in each cluster. (D, E) Expression distribution of marker genes in RB. The marker genes are shown on the left and the annotated cell types are shown on the right, the X axis represents cell clusters. (D). Genes are expressed in cell clusters. (E). Genes are not expressed in cell clusters.


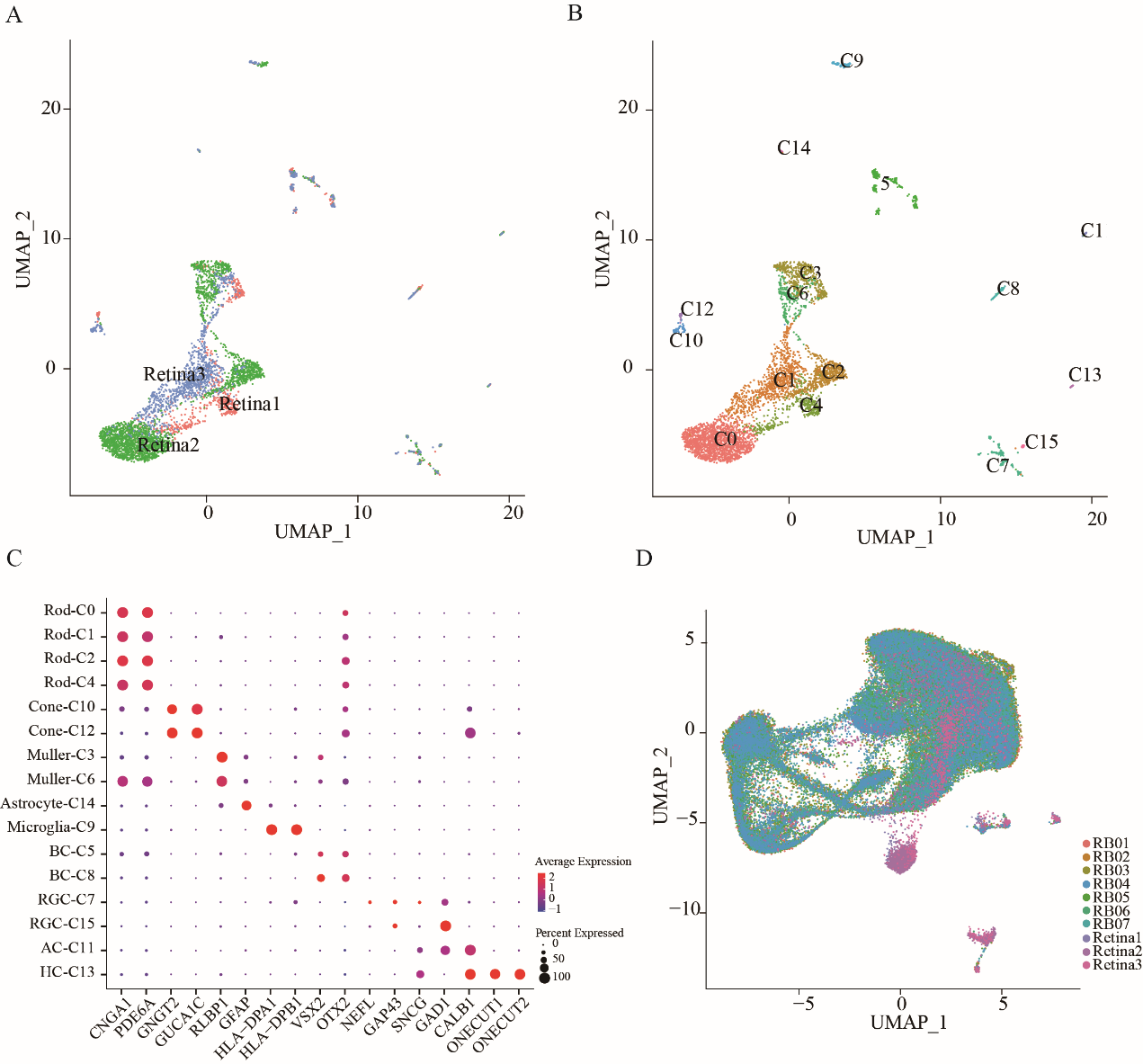


**Figure S3.** (A, B) U-MAP visualization of about 4000 retinal cells. Cells are colored by samples (A) and cell clusters (B). (C) Feature expression heatmap of a panel of known marker genes of retinal cell types. (D) U-MAP visualization of RB and retinal cells, colored by samples after integration through CCA.

­­­­
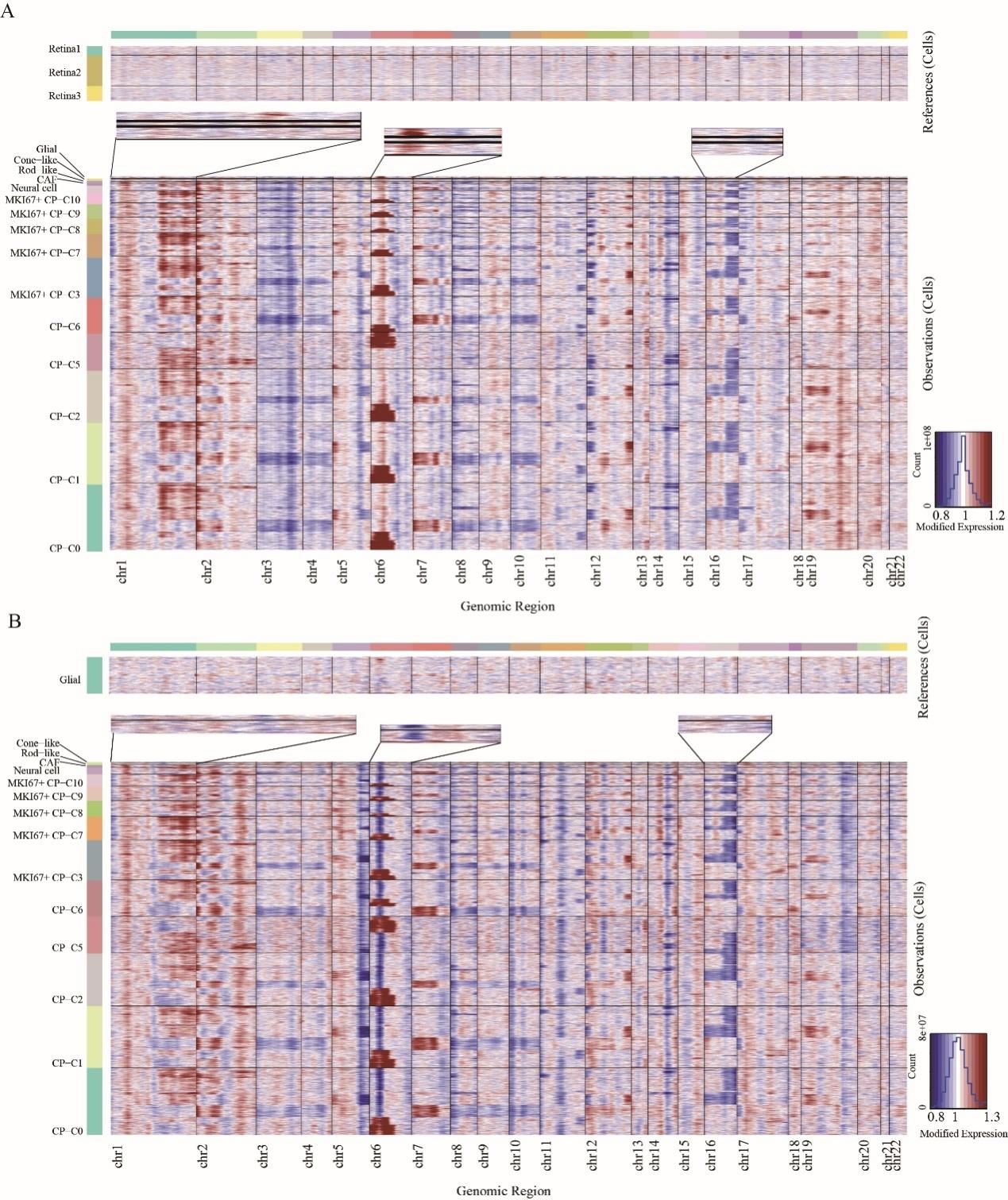


**Figure S4.** (A) CNV differences analysis the of different cell types in RB using normal retinal cells as a reference. **(B)** CNV differences analysis of different cell types in RB using glial cells as a reference. Copy number gains (red) and losses (blue).


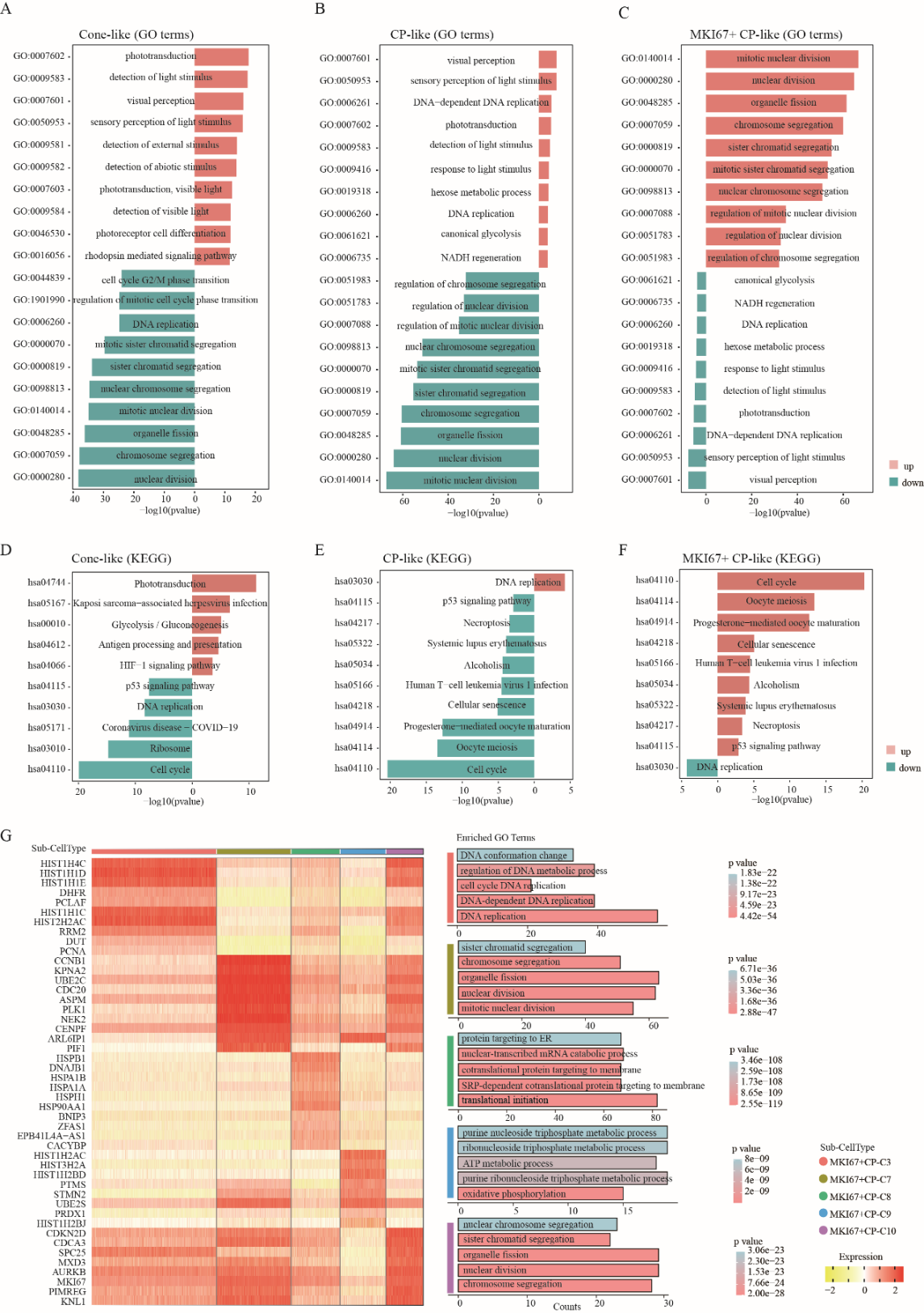


**Figure S5.** (A, B, C) The enriched gene ontology (GO) terms for genes with specific expression in cone-like (A) CP-like (B) and MKI67+ CP (C). (D, E, F) The enriched KEGG pathways for genes with specific expression in cone-like (D) CP-like (E) and MKI67+ CP (F). Red shows the function of up-regulated genes enrichment, and green shows the function of down-regulated genes enrichment. (G) Heatmap of significantly differentially expressed genes (left) and enriched gene ontology terms (right) of the MKI67+CP subtypes.


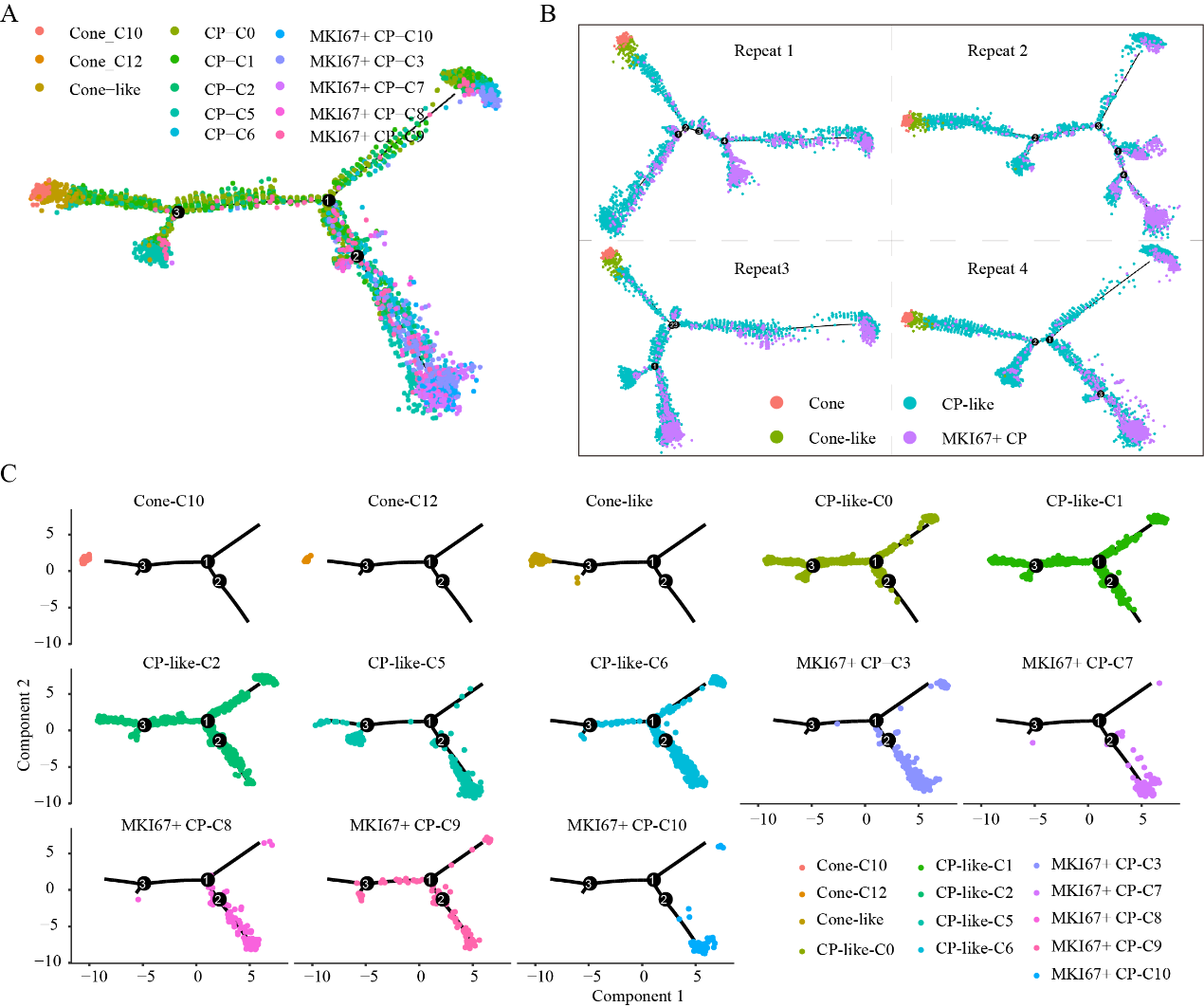


**Figure S6.** (A), Pseudo-time trajectory analysis of cone related cell subtypes, a point is a cell, colored by cell types. (B) Randomly selected cells for 4 repeated experiments. Each dot represents a single cell; the color of each dot indicates the cell type. (C) Cell subtypes were displayed on the pseudo-time trajectory.


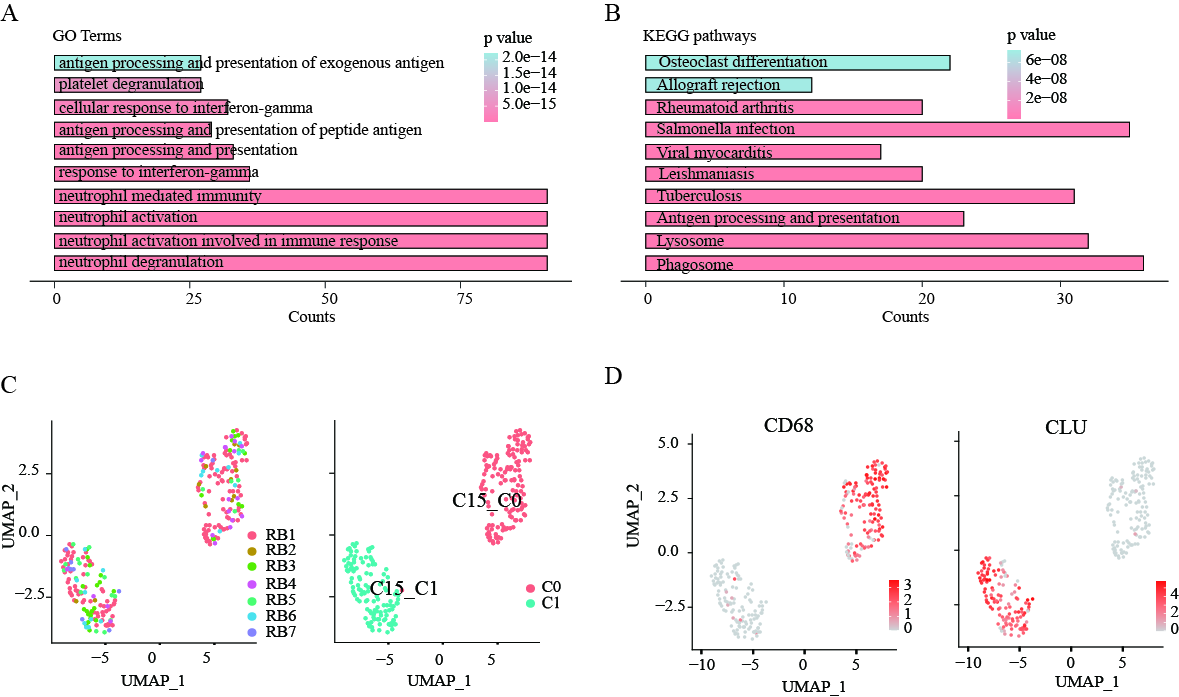


**Figure S7.** (A) Representative enriched GO terms in up-regulated genes of glial cells. (B) Representative enriched KEGG pathways in up-regulated genes of glial cells. (C) U-MAP visualization of glial cells. Cells are colored by samples and cell clusters. (D) Expression patterns of known markers (CD68, CLU) for macrophage and astrocyte on t-SNE plots. Grey to red indicates a gradient from low to high gene expression.


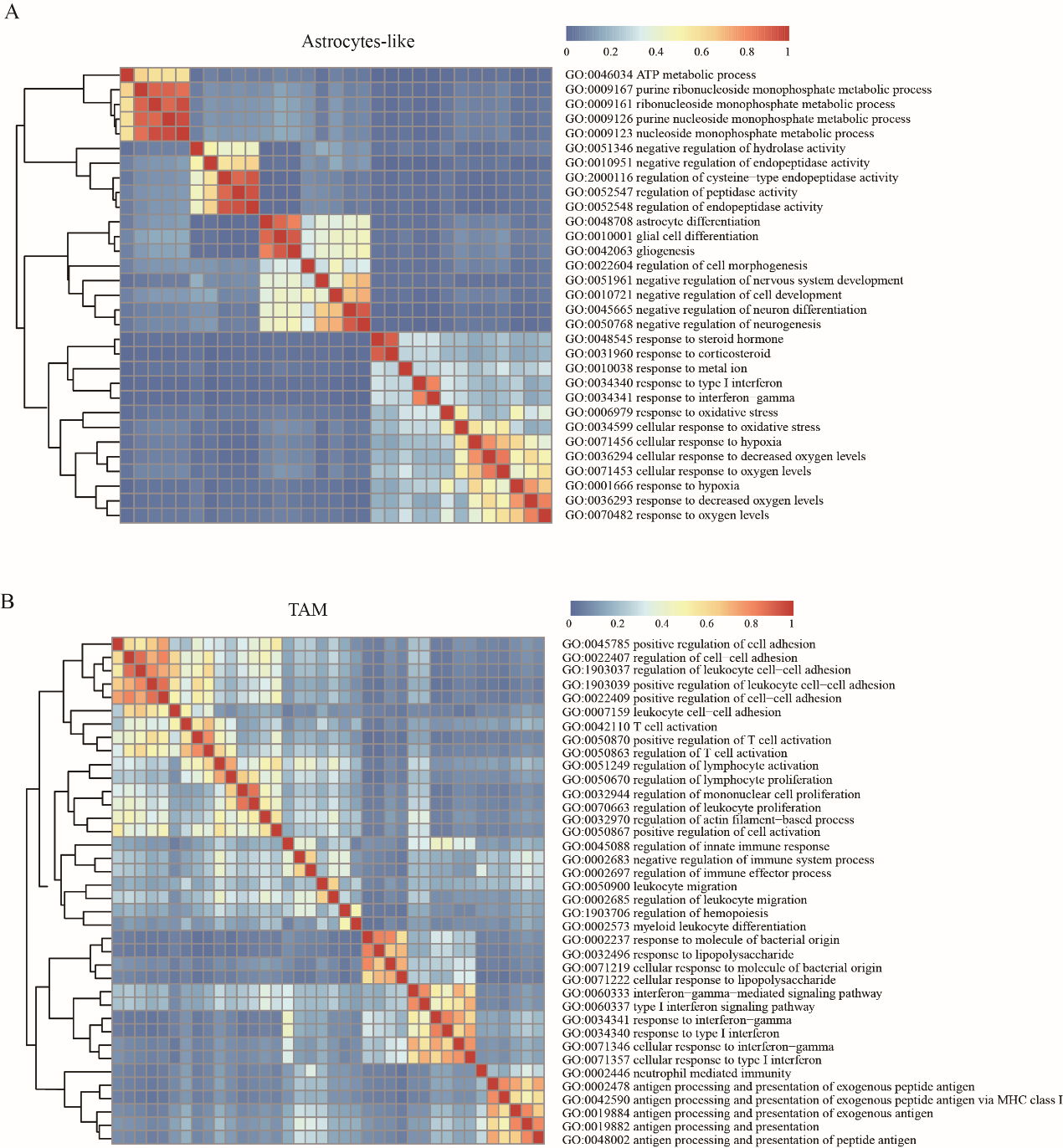


**Figure S8.** (A) Functional clustering heat map related to astrocyte-like. (B) Functional clustering heat map related to TAM.


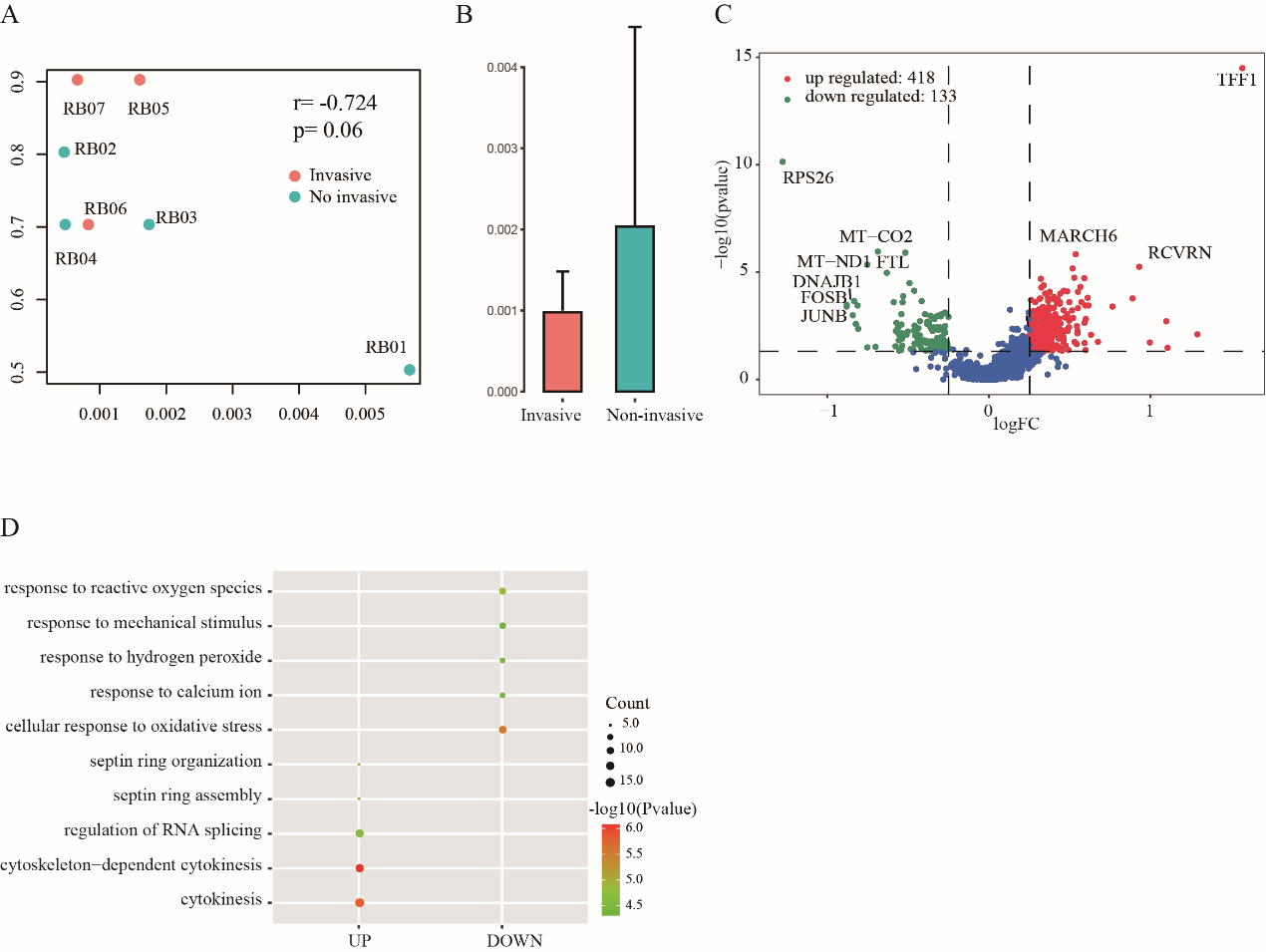


**Figure S9.** (A) Scatter plot of the proportion of astrocyte-like cells in different samples. (B) Histogram of the proportion of astrocyte-like cells in different samples. (C) Volcano plot for differential gene expression in invasive and non-invasive samples. (D) Functional differences of astrocyte-like cells between invasive and non-invasive samples.


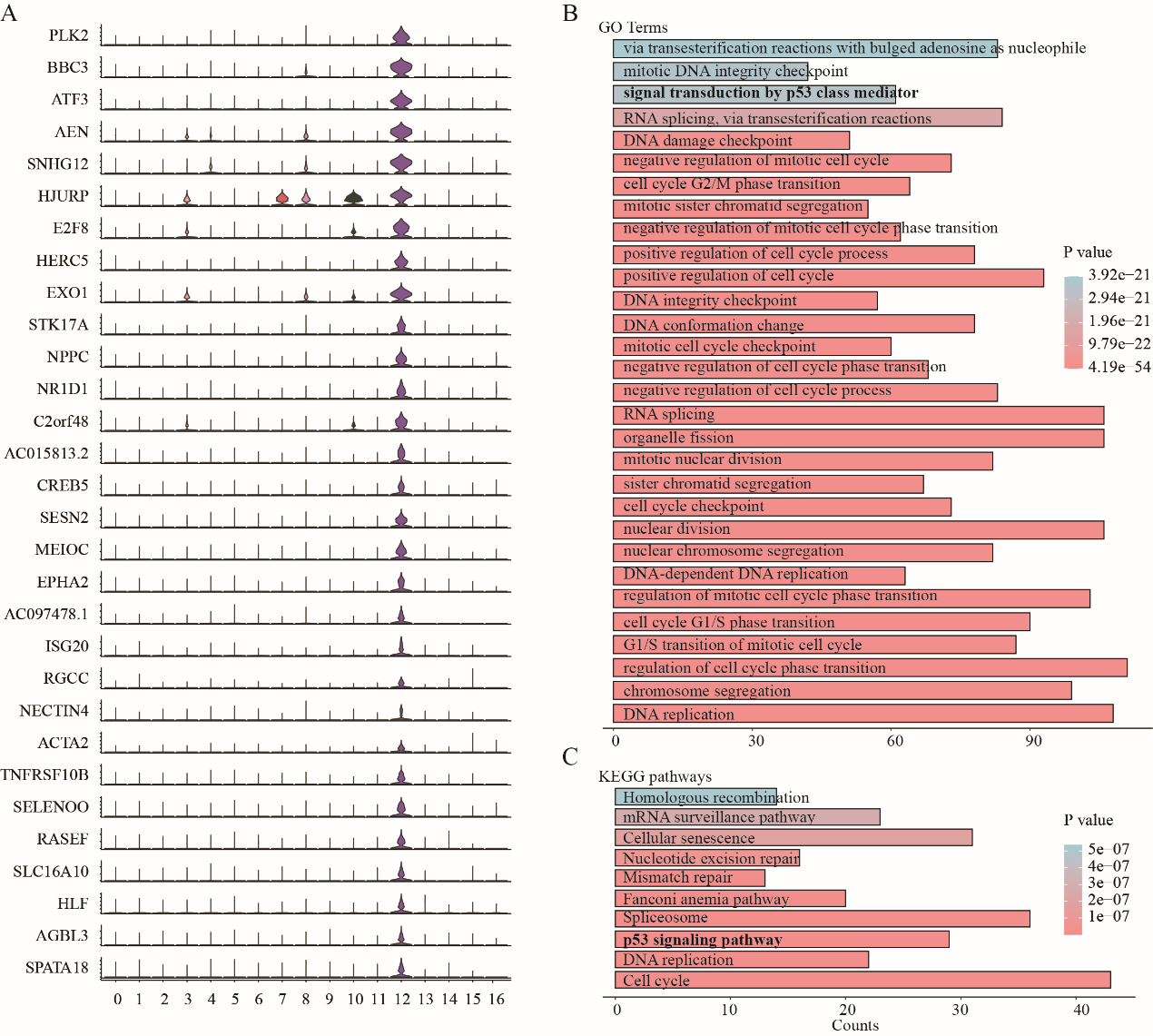


**Figure S10.** (A) Top 30 differentially expressed genes in every cell clusters. (B) Top 30 enriched GO terms in up-regulated genes of CAFs. (C) Top10 enriched KEGG pathways in up-regulated genes of CAFs.


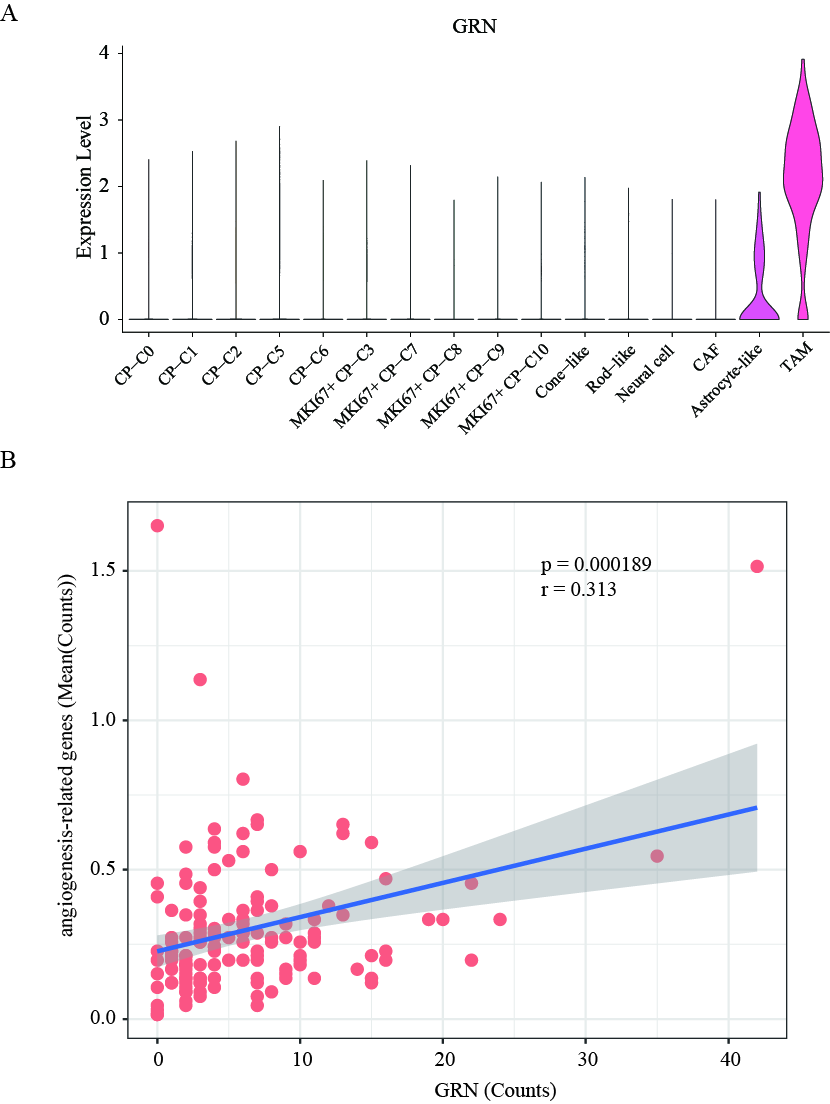


**Figure S11.** (A) Violin plots showing the distribution of GRN expression in different cell types. (B) Dot plot showing the correlation of GRN with angiogenesis-related genes (66 genes included, average expression) in TAMs.

**
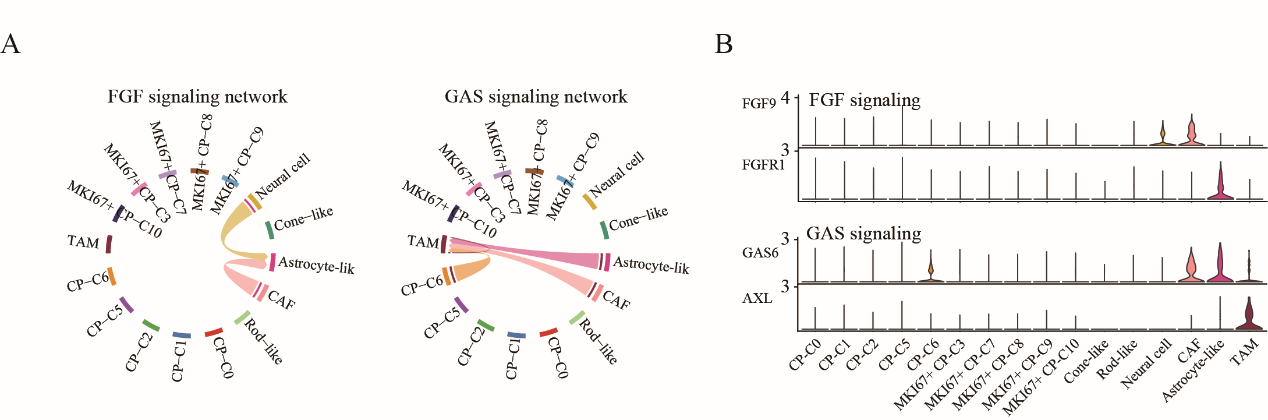
**

**Figure S12.** (A) This chord diagram shows CAF mediated signaling pathway networks, including FGF and GAS signaling networks. (B) Violin plot shows the expression of the FGF and GAS signaling pathway related ligands and receptors across 16 cell types.

**Table S1**

Details for donor retinoblastoma samples (n=15).

| **Sample** | **Sex** | **Right/ left** | **Age** | | **Blood Mut(RB1)** | **Histo pathology** | **Invasivenes** | **Ki67** | **Platform** |
| --- | --- | --- | --- | --- | --- | --- | --- | --- | --- |
| **RB01** | Female | Right | 7 Y | | No | No invasion of choroid and optic nerve. | Non-Invasive | 50%+ | 10X |
| **RB02** | Female | Left | 3Y 5M | | No | No invasion of choroid and optic nerve. | Non- Invasive | 80%+ | 10X&WES |
| **RB03** | Female | Right | 2Y 3M | | No | No invasion of choroid and optic nerve. | Non-Invasive | 70%+ | 10X |
| **RB04** | Male | Left | 2Y 6M | | No | No invasion of choroid and optic nerve. | Non-Invasive | 70%+ | 10X |
| **RB05** | Female | Right | 1Y 9M | | No | The tumor invaded a little choroid, and optic nerve. | Invasive | 90%+ | 10X |
| **RB06** | Male | Right | 1Y 8M | | No | The tumor invaded a little optic nerve and no invasion of choroid. | Invasive | 70%+ | 10X |
| **RB07** | Female | Left | 1Y 6M | | No | The tumor invaded the optic nerve and no invasion of choroid. | Invasive | 90%+ | 10X |
| **RB08** | Male | left | 2Y 7M | | No | The tumor invaded the choroid and optic nerve. | Invasive | 70%+ | WES |
| **RB09** | Male | left | 2Y | | No | The tumor invaded optic nerve and a little choroid. | Invasive | - | WES |
| **RB10** | Male | left | 1Y 9M | | No | No invasion of choroid and optic nerve. | Non-Invasive | 60%+ | WES |
| **RB11** | Male | left | 1 Y | No | | The tumor invaded a little cribriform and choroidal tissue. | Invasive | 80%+ | WES |
| **RB12** | Male | right | 2Y 5M | No | | The tumor invaded optic nerve. | Invasive | - | IHC |
| **RB13** | Female | right | 4Y 6M | No | | The tumor optic nerve. | Invasive | - | IHC |
| **RB14** | Female | left | 2Y 2M | No | | No invasion of choroid and optic nerve. | Non-Invasive | - | IHC |
| **RB15** | Male | right | 5Y | No | | No invasion of choroid and optic nerve. | Non-Invasive | - | IHC |

**Table S2**

Number of cells and genes per sample. RB01, RB02 and RB03 carried out technically repeats.

| **Sample** | **Number of clean reads** | **Estimated number of**  **cells** | **Estimated number of median genes** | **Filter**  **number of cells** | **Filter**  **number of median genes** |
| --- | --- | --- | --- | --- | --- |
| **RB01-rep1** | 216,851,227 | 6,990 | 2,689 | 5,840 | 2,765 |
| **RB01-rep2** | 200,088,809 | 6,826 | 2,605 | 5,736 | 2,680 |
| **RB02-rep1** | 194,597,113 | 4,185 | 1,938 | 3,345 | 1,968 |
| **RB02-rep2** | 236,740,694 | 2,140 | 1,692 | 1,509 | 1,816 |
| **RB03-rep1** | 192,752,092 | 7,596 | 1,949 | 5,420 | 2,125 |
| **RB03-rep2** | 198,492,229 | 7,638 | 1,895 | 5,835 | 2,007 |
| **RB04** | 439,897,029 | 14,093 | 2,338 | 9,384 | 2,561 |
| **RB05** | 368,513,039 | 13,016 | 2,435 | 9,670 | 2,489 |
| **RB06** | 370,652,458 | 14,407 | 2,118 | 11,629 | 2,235 |
| **RB07** | 536,603,867 | 14,681 | 2,447 | 11,452 | 2,557 |

**Table S3**

Marker genes of different cell types in the retina.

| **Cell Type** | **Marker genes** | **Reference** |
| --- | --- | --- |
| Retinal progenitor cells (RPC) | *SOX2, HES1, SFRP2, MKI67, HES5, FZD5, PAX6* | (1-5) |
| Pigment epithelium cells (PEC) | *SERPINF1, MITF, RPE65, BEST1, TTR* |  |
| photoreceptor precursor cells (PPC) | *CRX, RXRG, THRB, NRL* |  |
| Rods | *RHO, PDE6A, CNGA1, NRL, GNAT1, GNB1, SAG, ELOVL4, PDE6B, GNGT1* |  |
| Cones | *ARR3, GNGT2, PDE6H, GUCA1C, OPN1LW, GNAT2, RXRG, THRB, PDC, GNB3, CRX* |  |
| Horizontal cells (HC) | *ONECUT1, ONECUT2, ONECUT3, LHX1, TFAP2B* |  |
| Bipolar cells (BC) | *VSX2, VSX1, TRPM1* |  |
| Amacrine cells (AC) | *GAD1, CALB1, NRXN2, TFAP2A,* *PROX1, GAD2* |  |
| Retinal ganglion cells (RGC) | *POU4F2, GAP43, NEFL, SNCG, ATOH7, EBF3, THY1, NRN1* |  |
| Microglia cells | *HLA-DPA1, HLA-DPB1, HLA-DRA, C1QA, AIF1* |  |
| Astrocytes | *CLU, GFAP* |  |
| Muller glia cells | *CLU, RLBP1, PLP1, SOX2, SOX9, GLUL, APOE* |  |
| Fibroblasts | ACTA2*,* *COL1A1* |  |
| Retinal vasculature cells (RVC) | *CD34, CDH5* |  |

**Table S4**

Different cell types in human RB.

| **Cell Type** | | | **Cluster** | **Cells number** | **Marker Genes** |
| --- | --- | --- | --- | --- | --- |
| RB1+ | Glial cells | Macrophages | C15 | 138 | CD68, CD74, HLA-DRA, HLA-DRB1, HLA-DPA1, C1QB, C1QA |
|  |  | Astrocytes | C15 | 121 | CLU, GFAP, PAX2, GPX3, HES1, SOX2 |
|  |  | Fibroblasts | C12 | 414 | ACTA2, FGF9, VIM |
| RB1- | Mature photoreceptors | Rod-like | C13 | 394 | RHO, PDE6A, NRL, CNGA1, GNAT1, PDE6B, GNGT1, CRX, RXRG, THRB |
|  |  | Cone-like | C16 | 157 | PDE6H, GNGT2, ARR3, GUCA1C, GNAT2, CRX, RXRG, THRB |
|  | photoreceptor precursors | CP | C0, C1, C2, C5, C6 | 42692 (61%) | CRX, RXRG, THRB, MKI67(-) |
|  |  | MKI67+CP | C3, C7, C8, C9, C10 | 17731 (25%) | CRX, RXRG, THRB, MKI67(+), KIF14(+) |
|  |  | Neural cells | C11 | 1100 | CNTN2, GAP43, MAP2 |
|  |  | Others | C4, C14 | 7073 | RPL*, RPS* |

**Table S5**

Signature gene lists for M1-type, M2-type and tumor vascularization.

| **Signature** | **Genes** | **Reference** |
| --- | --- | --- |
| M1 signature | *NOS2, FCGR1A, CXCL10, CXCL11, CD86, TNF, CCL5, IRF5, IRF1, CD40, KYNU* | (6, 7) |
| M2 signature | *IL10, CD163, FCER2, CD274, MARCO, CSF1R, MRC1, IL1RN, IL4R, CCL4, CCL18, VEGFA, VEGFB, VEGFC, VEGFD, EGF, CTSA, CTSB, CTSD, TGFB1, TGFB2, TGFB3, MMP14, MMP19, MMP9, CLEC7A, TNFSF12, FN1* |  |
| Tumor vascularization | *VEGFA,KDR,FLT1,FGF2,HIF1A,ANGPT2,ADGRB1,THBS1,VEGFC,AKT1,FLT4,MMP2,ENG,MMP9,NOS3,KRIT1,PDCD10,NRP1,SERPINF1,PGF,AGGF1,HGF,CXCL8,PECAM1,DLL4,NDP,ADGRB2,ADGRB3,FZD4,ACE,COL18A1,TGFB2,SOD2,TIE1,VEGFD,TGFB1,CTNNB1,VEGFB,CCM2,LRP5,TIMP2,TYMP,PTGS2,ITGAV,TIMP1,VTN,IL1RN,CCL2,TIMP3,FGF1,HPSE,EPHB4,PIK3CA,ITGB1,FGFR2,KRAS,BAIAP2,HFE,TP53,CXCL12,PDGFRB,NRP2,NOTCH1,VWF,BMP6,ITGB3* | ^(8)^ |

**Reference**

1. Hu Y, Wang X, Hu B, Mao Y, Chen Y, Yan L, et al. Dissecting the transcriptome landscape of the human fetal neural retina and retinal pigment epithelium by single-cell RNA-seq analysis. PLoS Biol. 2019;17(7):e3000365.

2. Lukowski SW, Lo CY, Sharov AA, Nguyen Q, Fang L, Hung SS, et al. A single-cell transcriptome atlas of the adult human retina. EMBO J. 2019;38(18):e100811.

3. Menon M, Mohammadi S, Davila-Velderrain J, Goods BA, Cadwell TD, Xing Y, et al. Single-cell transcriptomic atlas of the human retina identifies cell types associated with age-related macular degeneration. Nat Commun. 2019;10(1):4902.

4. Sridhar A, Hoshino A, Finkbeiner CR, Chitsazan A, Dai L, Haugan AK, et al. Single-Cell Transcriptomic Comparison of Human Fetal Retina, hPSC-Derived Retinal Organoids, and Long-Term Retinal Cultures. Cell Rep. 2020;30(5):1644-59 e4.

5. Voigt AP, Whitmore SS, Flamme-Wiese MJ, Riker MJ, Wiley LA, Tucker BA, et al. Molecular characterization of foveal versus peripheral human retina by single-cell RNA sequencing. Exp Eye Res. 2019;184:234-42.

6. Navarro-Barriuso J, Mansilla MJ, Martinez-Caceres EM. Searching for the Transcriptomic Signature of Immune Tolerance Induction-Biomarkers of Safety and Functionality for Tolerogenic Dendritic Cells and Regulatory Macrophages. Front Immunol. 2018;9:2062.

7. Zheng Y, Chen Z, Han Y, Han L, Zou X, Zhou B, et al. Immune suppressive landscape in the human esophageal squamous cell carcinoma microenvironment. Nat Commun. 2020;11(1):6268.

8. Zhen Z, Shen Z, Hu Y, Sun P. Screening and identification of angiogenesis-related genes as potential novel prognostic biomarkers of hepatocellular carcinoma through bioinformatics analysis. Aging (Albany NY). 2021;13(13):17707-33.
